# Supplementary material for: Core outcome set for preventive intervention trials in chronic and episodic migraine (COSMIG): an international, consensus-derived and multistakeholder initiative
Source: BMJ Open. 2021 Nov 30;11(11):e043242. doi: 10.1136/bmjopen-2020-043242 (PMC8634270; doi:10.1136/bmjopen-2020-043242)
Supplement: Supplementary data [file bmjopen-2020-043242supp001.pdf]

APPENDIX

Appendix Table 1. Bespoke grading system to illustrate where consensus was achieved in the Delphi Round 1 for reviewed domains.

Grade	Level of agreement between panel	Decision rule
A **	If in both panels the median rating is 9	Include domain in Round 2
A*	If in both panels ≥70% rate a domain ≥7	Include domain in Round 2
A	If in both panels the median domain rating is ≥7	Include domain in Round 2 if either panel achieves a median score of 9 OR qualitative evidence supports further consideration
B	If the median rating for a domain is ≥7 in only one panel	Include domain in Round 2 if either panel achieves a median score of 9 OR qualitative evidence supports further consideration
C	If the median rating for the two panels combined is ≥4 and ≤6 and the median rating for no single panel is ≤7	No progression to Round 2 (unless qualitative evidence supports further consideration)
D	If the median rating for the two panels combined is ≥1 and ≤3 and the median rating for no single panel is ≤7	No progression to Round 2 (unless qualitative evidence supports further consideration)

Footnote: ‘both panels’ refers to – patient panel and professionals panel

Haywood K, *et al. BMJ Open* 2021; 11:e043242. doi: 10.1136/bmjopen-2020-043242

Appendix Table 2. Background of professional participants (expert panel) in the Delphi process (Round 1).

	Chronic round	Episodic round
Clinician	6	5
Neurologist	13	12
Neurologist specialist interest headache	10	11
GP specialist interest headache	1	0
Nurse specialist	4	3
Chiro/osteopath/	2	1
Health Economist	2	1
Clinical Academic	8	9
Other health professional academic	2	0
Clinical Trialist	9	8
Systematic reviewer	6	5
Measurement expert	7	8

Footnote: participants could identify as having more than one background

Appendix Table 3. Delphi Round 3: results of voting on sub-panel discrepancies.

Outcome to be voted on (R3)			EPISODIC MIGRAINE *Voting*			CHRONIC MIGRAINE *Voting*		
Discrepancies (outcomes rated in top 50% by one sub-panel)	Proposed Domain and definition	Q	Patient (n=23)	HCPs (n=21)	Combined (n=44)	Patient (n=29)	HCPs (n=23)	Combined (n=52)
Ranked highly by healthcare professionals (HCPs)								
• *HCP 9/27; Patients 20/27 (EM)*	• **Satisfaction with Treatment**	a.	65.2%	**71.4%**	68.2%	-	-	-
• *HCP 8/27; Patients 25/27 (EM)*	• **Vomiting and/ feelings of nausea**	a.	60.9%	**71.4%**	65.9%	-	-	-
• *HCP 12/27; Patients 18/27 (EM)*	• Type (potency) and dose (how much) of a medication when experiencing a migraine	a.				-	-	-
• *HCP 10/31; Patients 20/31 (CM)*	• Stress – feelings of distress, frustration or irritation	a.	-	-	-	58.6%	47.8%	53.9%
• *HCP 15/31; Patients 29/31 (CM)*	• Mortality (death)	a.	-	-	-	20.7%	17.4%	19.2%
Ranked highly by patients								
• *Patients 10/27; HCPs 21/27 (EM)*<br>• *Patients 14/31; HCPs 31/31 (CM)*	• **Unpredictability of a migraine – uncertainty of being symptom-free or able to engage in activities**	a.	**82.6%**	61.9%	**72.7%**	**96.6%**	**69.6%**	**84.6%**
• *Patients 11/27; HCPs 23/27 (EM)*	• **Physical fatigue – experiencing physical fatigue, tiredness, lacking in energy, feeling physically exhausted**	a.	**69.6%**	52.4%	61.4%	-	-	-
• *Patients 10/27; HCPs 21/27 (EM)*	• **Depressive mood – feeling sad, feeling down, feeling sorry for oneself, or feeling depressed**	a.	**69.6%**	42.9%	56.8%	-	-	-

Footnote: Panellists were asked to indicate (Yes/No): a. Should the following outcomes be included in a core set for studies of EM / CM (respectively)?
