# Supplementary material for: Core outcome set for preventive intervention trials in chronic and episodic migraine (COSMIG): an international, consensus-derived and multistakeholder initiative
Source: BMJ Open. 2021 Nov 30;11(11):e043242. doi: 10.1136/bmjopen-2020-043242 (PMC8634270; doi:10.1136/bmjopen-2020-043242)
Supplement: Author's manuscript [file bmjopen-2020-043242.draft_revisions.pdf]

BMJ Open

BMJ Open is committed to open peer review. As part of this commitment we make the peer review history of every article we publish publicly available.

When an article is published we post the peer reviewers' comments and the authors' responses online. We also post the versions of the paper that were used during peer review. These are the versions that the peer review comments apply to.

The versions of the paper that follow are the versions that were submitted during the peer review process. They are not the versions of record or the final published versions. They should not be cited or distributed as the published version of this manuscript.

BMJ Open is an open access journal and the full, final, typeset and author-corrected version of record of the manuscript is available on our site with no access controls, subscription charges or pay-per-view fees (http://bmjopen.bmj.com).

If you have any questions on BMJ Open's open peer review process please email info.bmjopen@bmj.com

BMJ Open

A Core Outcome Set for Preventive Intervention Trials in Chronic and Episodic Migraine (COSMIG): An international, consensus-derived and multi-stakeholder initiative.

Journal:	*BMJ Open*
Manuscript ID	bmjopen-2020-043242
Article Type:	Original research
Date Submitted by the Author:	12-Oct-2020

Complete List of Authors:	Haywood, Kirstie; University of Warwick Warwick Medical School, Warwick Research in Nursing<br>Potter, Rachel; University of Warwick Warwick Medical School, Clinical Trials Unit<br>Froud, Robert; Kristiania University College, Institute of Health Sciences<br>Pearce, Gemma; Coventry University Department of Psychology and Behavioural Sciences<br>Box, Barbara; University of Warwick Warwick Medical School, Clinical Trials Unit<br>Muldoon, Lynne; University of Warwick, Clinical Trials Unit, Warwick Medical School<br>Lipton, Richard; Albert Einstein College of Medicine Department of Neurology, Department of Neurology<br>Petrou, Stavros; Nuffield Department of Primary Care Health Sciences,<br>Rendas-Baum, Regina; QualityMetric, Outcomes Insight Consulting<br>Logan, Anne-Marie; St George's University Hospitals NHS Foundation Trust<br>Stewart, Kimberley; University of Warwick Warwick Medical School, Clinical Trials Unit<br>Underwood, Martin; University of Warwick Warwick Medical School, Clinical Trials Unit<br>Matharu, Manjit; University College London Hospitals NHS Foundation Trust National Hospital for Neurology and Neurosurgery, The Headache Group

Keywords:	Migraine < NEUROLOGY, STATISTICS & RESEARCH METHODS, Clinical trials < THERAPEUTICS

SCHOLARONE™ Manuscripts

I, the Submitting Author has the right to grant and does grant on behalf of all authors of the Work (as defined in the below author licence), an exclusive licence and/or a non-exclusive licence for contributions from authors who are: i) UK Crown employees; ii) where BMJ has agreed a CC-BY licence shall apply, and/or iii) in accordance with the terms applicable for US Federal Government officers or employees acting as part of their official duties; on a worldwide, perpetual, irrevocable, royalty-free basis to BMJ Publishing Group Ltd ("BMJ") its licensees and where the relevant Journal is co-owned by BMJ to the co-owners of the Journal, to publish the Work in this journal and any other BMJ products and to exploit all rights, as set out in our licence.

The Submitting Author accepts and understands that any supply made under these terms is made by BMJ to the Submitting Author unless you are acting as an employee on behalf of your employer or a postgraduate student of an affiliated institution which is paying any applicable article publishing charge ("APC") for Open Access articles. Where the Submitting Author wishes to make the Work available on an Open Access basis (and intends to pay the relevant APC), the terms of reuse of such Open Access shall be governed by a Creative Commons licence – details of these licences and which Creative Commons licence will apply to this Work are set out in our licence referred to above.

Other than as permitted in any relevant BMJ Author's Self Archiving Policies, I confirm this Work has not been accepted for publication elsewhere, is not being considered for publication elsewhere and does not duplicate material already published. I confirm all authors consent to publication of this Work and authorise the granting of this licence.

For peer review only

A Core Outcome Set for Preventive Intervention Trials in Chronic and Episodic Migraine (COSMIG): An international, consensus-derived and multi-stakeholder initiative.

Haywood KL, Potter R, Froud R, Pearce G, Box B, Muldoon L, Lipton RB, Petrou S, Rendas-Baum R, Logan A, Stewart K, Underwood M, Matharu M; on behalf of the CHESS COSMIG group.

Corresponding author: Dr Rachel Potter r.potter@warwick.ac.uk

Main Text (3,651/4000)

Figure 1. Flow diagram outlining the development stages for the COSMIG
Figure 2. COSMIG: Core Outcome Set for Episodic and Chronic MIGraine.

Table 1. Delphi Round 1 Shortlisted domains
Table 2a. Delphi Round 2. Results of domain prioritisation for Episodic Migraine (combined panels)
Table 2b. Delphi Round 2. Results of domain prioritisation for Chronic Migraine (combined panels)
Table 3. Delphi Round 3. Results of voting for domains for episodic and chronic migraine
Table 4. Consensus meeting: results from small and large group discussions and voting.

Appendix

Appendix Table 1. Grading system
Appendix Table 2. Delphi participants - professional background
Appendix Table 3. Delphi Round 3. Results of voting on sub-panel discrepancies.

For peer review only

For peer review only - http://bmjopen.bmj.com/site/about/guidelines.xhtml

ABSTRACT (249//300 words)

Objective: Typically, migraine prevention trials focus on reducing migraine days. This narrow focus may not capture all that is important to people with migraine. Inconsistency in outcome selection across trials limits the potential for data pooling and evidence synthesis. In response, we describe the development of core outcome set for migraine (COSMIG).

Design: A two-stage approach sought to achieve international, multi-stakeholder consensus on both the core domain set (CDS) and core measurement set (CMS). Following construction of a comprehensive list of outcomes, expert panellists (patients, healthcare professionals and researchers) completed a three-round electronic-Delphi study to support a reduction and prioritisation of core domains and outcomes. Participants in a consensus meeting finalised the core domains and methods of assessment. All stages were overseen by an international core team, including patient research partners.

Results: There was good representation of patients (episodic (n=34) and chronic migraine (n=42)) and healthcare professionals (n=33) with high response and retention rates. The initial list of domains and outcomes was reduced from >50 to seven core domains for consideration in the consensus meeting, during which a two-domain core outcome set was agreed.

Conclusion: International and multi-stakeholder consensus emerged to describe a two-domain core outcome set for reporting research on preventive interventions for chronic and episodic migraine: migraine-specific pain and migraine-specific quality of life. Intensity of migraine-pain assessed with an 11-point numerical rating scale and the frequency as the number of headache/migraine days over a specified time-period. Migraine-specific quality of life assessed using the Migraine Functional Impact Questionnaire.

Strengths and limitations of this study:

- The research process and validity of results are strengthened by the co-collaboration with patient research partners throughout all stages of the research.
- A bespoke grading system to support the prioritisation of outcome domains between stakeholder groups (expert panels) is described.
- International, multi-stakeholder participation – patients, researchers and a range of health professionals - in the on-line Delphi survey.
- Expert panel representation in the Delphi survey was largely from Europe and North America.
- The majority of participants in the face-to-face consensus meeting were from the UK.

For peer review only - http://bmjopen.bmj.com/site/about/guidelines.xhtml

BACKGROUND

International guidelines for the conduct of preventive studies for both episodic and chronic migraine specify that the primary outcome should be focussed on migraine days, or for chronic migraine on moderate to severe headache days [1]. Reviews of clinical trials of populations with chronic migraine and episodic migraine have identified substantial inconsistencies in outcomes reporting alongside often poorly defined outcomes [2, 3]. An important impact of these inconsistencies is to limit the potential for robust meta-analyses [4 5]. For example, a 2015 meta-analysis of drugs for the prophylaxis of migraine by Jackson et al [6] did not include data from the largest and most robust trial of topiramate for chronic migraine (n=307) that found a mean difference of 1.7 migraine/migrainous days per 28 days after 12 weeks [7]. The reviewers meta-analysed the data from two much smaller (n=32 & n=50), low quality studies, and reported an effect size of 8.4 headache days, the outcome specified for the meta-analyses, after 12 weeks. Data that cannot be interpreted or utilised can result in unacceptable and unethical research waste. There is also potential for selective outcomes reporting and associated reporting bias if consistent outcomes are not pre-specified [8, 9].

Improved consistency, accountability and transparency in outcome reporting can be achieved by using a Core Outcome Set (COS); a small, standardised group of outcomes that should be measured and reported, as a minimum, in all effectiveness trials for a specific health area [10-12].
Current international guidelines for conduct of prevention studies in episodic or chronic migraine have not developed outcome reporting recommendations in line with current best practice [1, 13]. Notably, patient input is markedly absent from these guidelines.
We describe here the development of a multiple-stakeholder, internationally endorsed, consensus-based COS applicable to preventative intervention trials and research studies in adults with episodic or chronic MIGraine (COSMIG).

Methods

Two key stages in core outcome set development are described (figure 1) [14]:

Stage 1) Defining the core domain set: WHAT to measure; i.e., the minimum number of health domains that must be assessed. A domain describes the concept or 'aspect of health or a health condition that needs to be measured to appropriately assess the effects of a health intervention' [14].

Stage 2) Recommending the core measurement set: HOW to measure, i.e., the minimum set of assessment methods that adequately correspond to the core domain set.

For peer review only - http://bmjopen.bmj.com/site/about/guidelines.xhtml

We prospectively registered COSMIG with the Core Outcomes Measures in Effectiveness Trials (COMET) initiative [http://www.comet-initiative.org/studies/details/953?result=true]. Ethical approval was gained from Warwick Medical School Biomedical and Scientific Research Ethics Committee REGO-2017-1921.

Patient and public involvement

Following good practice guidance [https://www.invo.org.uk/posttyperesource/before-you-start-involving-people/; [15], we worked collaboratively with our patient research partners throughout all stages of the research.

The COSMIG core group consisted of clinicians with expertise in headaches and migraine (MM,MU, BD, RL,RJ), research scientists with expertise in clinical trials, health measurement and qualitative research (MU,KH,RF,RP,SP,VN,SP,KS) and patient research partners (GP,BB,LM).. Regular meetings were held between all group members, and specifically between each Delphi round, to discuss results, confirm feedback and format for subsequent rounds.

Stage 1 Core Domain Set

Stage 1.1: Developing a comprehensive domain list

We first identified potential domains from systematic reviews [2, 3] and qualitative research [16]. Domains were written in plain English as on-line questionnaires: one questionnaire contained domains for episodic headache, and one for chronic headache. Questionnaires were piloted with the core team and researchers naïve to the study (n=12).

Stage 1.2: International modified-Delphi process

Our primary goal, for our Delphi study was to refine and prioritise domains. We sought consensus between experts on the core domain set. We defined two expert panels external to the core research team: one comprised of patients with a target of 50 with chronic migraine (CM) and 50 with episodic migraine (EM); and a second panel (also with a target of 50) comprised of healthcare professionals and researchers.

Patients: We asked 13 national/international organisations to advertise the study on their social media platforms (Appendix 1). Interested participants (≥18-years old) contacted the research team. We asked participants to self-diagnose/classify their migraines as episodic or chronic migraine. Patient participants completed episodic *or* chronic migraine questionnaires depending on their self-diagnosis.

For peer review only - http://bmjopen.bmj.com/site/about/guidelines.xhtml

Professionals: We invited national and international healthcare professionals (neurologists, GPs, nurses, psychologists, pharmacists, allied health professionals) and researchers (trialists, reviewers, health economists, measurement experts) involved in headache research identified through professional societies and from published research to participate. They were asked to complete *both* questionnaires.

The Delphi process had three sequential rounds with participants completing each prior round eligible to complete the next. The Delphi study administration and hosting of the on-line questionnaires was managed by Clinvivo Ltd.

For peer review only

Round 1 Participants rated the relative importance of each domain for inclusion in future research studies of chronic or episodic headache using a nine point numerical rating scale (range 1 to 3 'Not at all important', 4 to 6 'Uncertain', and 7 to 9 'Very important'). Participants could elaborate on their decisions and/or provide additional domains for consideration in subsequent rounds. Informed by an approach described by Orbai et al. (2017) [17], we devised a bespoke grading system to illustrate where consensus was achieved and to indicate more easily where participants in each group disagreed in their judgement (Appendix Table 1).

An *a priori* decision rule determined that only those outcome domains judged most favourably by one or both groups (patients and professionals) would be included in round two.

Round 2 In round two we focused more specifically on migraine-specific, rather than headache-specific, domains. Responses to round one were summarised and anonymous feedback provided (own score; group median scores). Further prioritisation was achieved by inviting participants to 'spend points' (up to a maximum of 70) to illustrate how strongly they felt that a domain should be prioritised for inclusion in the core domain set; a maximum of 10 points could be allocated to any one outcome domain (11-point scale, 0 'Not a priority' to 10 'Absolute priority'). To ensure that group differences were observed, the results from both groups were considered both separately and combined: the top 10 and top 50% of prioritised domains were discussed between COSMIG core team members, informing the maintenance of, or, where the concepts of health were similar, grouping of domains into a single 'meaningful' domain.

Round 3 Responses to round two were summarised, highlighting the top 50% of prioritised domains and between-group discrepancies. For those domains prioritised highly by just one group (top 50%), participants were asked to reconsider if they should be included in the priority listing. If more than 70% of respondents selected 'yes', the domain was included. Finally, participants were asked to

For peer review only - http://bmjopen.bmj.com/site/about/guidelines.xhtml

indicate by means of a dichotomous response if they: a) were happy with the grouping of prioritised domains; b) were happy with the proposed 'meaningful' domain and definition; and c) had additional comments. The frequency distribution of responses was calculated.

Stage 2: Core Measurement Set

International expert panel face-to-face meeting

The purpose of the one-day meeting was to confirm the core domain set developed in our Delphi study, agree the core measurement set, and recommend the core outcome set. Importantly, participants were to consider that whilst a domain may be considered important, if an acceptable approach to measurement is not available, it is not appropriate to include the domain in a core outcome set.

We invited professionals from Europe and patients from the UK who had taken part in our Delphi study. Participants received an information pack with meeting objectives and domain/measurement information ahead of the meeting. Where existing consensus for potential measures was not available, the COSMIG core team reviewed key data sources for guidance and evidence of measurement quality, acceptability and feasibility for use in preventive studies of episodic or chronic migraine:

- Migraine / headache:
    - Review of patient-reported outcome measures (PROMS)[2]
    - International Headache Society guidelines [1, 13, 18]
    - National Institute for Neurological Disorders Common Data Elements – Headache (preventative treatment)[19]
- Chronic Pain and core outcome set development
    - Initiative on Methods, Measurement and Pain Assessment in Clinical Trials[20-22]
    - Outcome Measures in Rheumatology and Clinical Trials group [23]

The meeting started with an overview of the results of the Delphi process, prioritised domains and the evidence-base underpinning potential methods of assessment. Participants considered three options when determining domain 'placement' within the final core outcome set [17]:

i) *Core 'inner' circle:* domain is unambiguous with an acceptable method of assessment;

ii) *Middle circle:* domain is important, but not feasible for all preventative trials and research studies;

For peer review only - http://bmjopen.bmj.com/site/about/guidelines.xhtml

iii) *Outer circle*: domain is important, but requires further study (research agenda) – e.g. lacks conceptual clarity or method of assessment.

Semi-structured, small-group discussions with a mix of patients, healthcare professionals/researchers and members of the core research team (including patient partners) ensued, covering each prioritised domain. Two facilitators each supported two rounds of discussion per domain. Outcome domains and methods of assessment were reviewed in terms of importance, quality, acceptability and feasibility. Facilitators supported participant contribution, sharing findings between groups to foster the flow of thinking. Following each small-group discussion, participants, with the exception of the core research team, were asked to indicate anonymously (paper-based questionnaire) their preference for domain inclusion (yes/no/don't know) and assessment (selecting one option from a short-list); where ≥ 70% of panellists agreeing was set as an *a priori* definition of agreement.

Next, small group discussions and results were presented to the whole group. Where there was agreement, no further discussion was required. Subsequent discussion focused on where further refinement was required. Finally, participants voted electronically to confirm domain placement in the COS (inner/middle/outer/out) and method of assessment. Proceedings were captured in the form of detailed written records and the outcomes of voting.

Results

Stage 1 Core Domain Set

Stage 1.1: Developing a comprehensive domain list

A total of 57 (episodic) and 58 (chronic) domains were included in the questionnaire, grouped across four areas: symptoms (17), life impact (27 episodic / 28 chronic), treatment effectiveness/ financial impact (10) and complications (2). Piloting informed minor language modifications. Fifty seven of the domains of interest were included for both episodic and chronic migraine.

Stage 1.2: International modified-Delphi process

<u>*Round one*</u>

Group 1 (patients) Two organisations advertised the study (Migraine Association, Ireland; National Migraine Centre, UK). Almost 80% (76/96) of patients who expressed an interest in taking part in the study completed the first questionnaire (42/53 CM (79%); 34/43 EM (79%)). Most were female (CM 40/53 (73%); EM 29/43 (66%)) and aged between 36-45 (CM 41%) and 56-65 years (EM 32%) (range

For peer review only - http://bmjopen.bmj.com/site/about/guidelines.xhtml

18 to >66 years). Most were from the UK (57%), followed by the US (19%), Ireland (14%), Canada (2%), and the rest of Europe (Denmark (2%), France (5%)).

Group 2 (professionals) From a total of 198 international healthcare professionals/researchers invited to participate, 64 agreed. Nearly half (31/64 (48%)) completed the episodic migraine questionnaire and slightly more (33/64 (52%)) completed the chronic migraine questionnaires. Most were from the UK 14/33 (42%), with participants from the US 5/33 (15%), Europe (Belgium 1/33 (3%), Germany 2/33 (6%), Italy 1/33 (3%), Netherlands 1/33 (3%), Portugal 1/33 (3%), Serbia 1/33 (3%), Spain 2/33 (6%)) and Turkey 1/33 (3%)), the Russian Federation 1/33 (3%), South Africa 1/33 (3%) and Thailand 1/33 (3%). Professionals included neurologists, nurse specialists, general practitioner, allied health professionals, researchers and measurement experts (Appendix Table 2).

In total, 75 (64%) and 65 (61%) panellists completed round 1 chronic and episodic migraine questionnaires, respectively.

Most domains were rated as 'important', with few between group discrepancies. Implementation of the *a priori* decision rule (Appendix Table 1) supported a 50% reduction in domains, with the prioritisation of 18/57 (episodic) and 24/58 (chronic) domains (Table 1). Qualitative feedback informed further consideration of 10 domains (9 episodic, 7 chronic) not achieving the proposed benchmark. No 'new' domains were proposed.

Table 1. Delphi Round 1 shortlisted domains by voting prioritisation and agreement between groups

Domain	EPISODIC MIGRAINE		CHRONIC MIGRAINE	
	Evidence supporting inclusion in Round 2 Delphi			
Section 1: Life impact – symptoms associated with headache / migraine	Voting prioritisation	Qualitative feedback	Voting prioritisation	Qualitative feedback
Cognitive function – difficulty concentrating, ability to 'think clearly' or to remember things	(A)	Yes	A*	
Increased sensitivities – to light, sound, smell, touch	A*		A*	
Pain associated with headache – experience an unpleasant physical sensation that aches or hurts	A**		A*	
Duration of pain associated with a headache	A**		A*	
Frequency of pain associated with a headache	A**		A*	
Severity / intensity of pain associated with a headache	A**		A*	
Physical fatigue – experiencing physical fatigue, tiredness, lacking in energy, feeling physically exhausted	(A)	Yes	A*	
Sleep quality – being able to have a restful sleep	(A)		A*	
Vomiting and/or feelings of nausea	A*		(A)	
Anxiety – concerned, worried, fearful or anxious	(A)	Yes	(A)	Yes
Depressive mood – feeling sad, feeling down, feeling sorry for oneself or feeling depressed	(A)	Yes	(A)	No

For peer review only - http://bmjopen.bmj.com/site/about/guidelines.xhtml
For peer review only

Section 2: Life impact – functioning, activities and general wellbeing				
Activities of daily life				
Being able to carry out ***usual tasks or daily activities*** inside or outside the home (not related to paid employment) ***that support an independent lifestyle*** – such as tidying one's home, walking short distances, managing finance, driving, using technology	(A)		A*	
Needing to rest or lie down ***because of*** a headache	(A)		A*	
Emotional wellbeing				
Feelings of isolation – feeling isolated, reduced social interactions	(B)	Yes	(A)	Yes
Self-worth – feeling like a burden to others; can include feeling valued or helpless, accepted or rejected; feelings of self-esteem	(B)	Yes	(A)	Yes
Stress – feelings of distress, frustration or irritation	A*		(A)	Yes
Work/Education				
Being able to carry out ***activities related to work*** (paid or unpaid) / ***study*** to an acceptable or usual standard	A*		A**	
Needing to take ***time-off work*** (paid or unpaid) / ***study***	A*		A*	
Social life				
Social life – relationships with colleagues or peers			A*	
Family roles – being able ***to provide*** usual care and support for family and close friends	(A)	Yes	(A)	Yes
Participation in social or leisure activities – ability to participate in social or leisure activities	(A)	Yes	(A)	Yes
Overall health – an individual's general health status; the ability to live a 'normal ' life	A*		A*	
Self-management – ability to effectively decrease/ minimise / control the impact of migraine on oneself (e.g. pharmacology, diet, lifestyle choices)	A*		A*	
Unpredictability of a migraine – uncertainty of being symptom-free or able to engage in activities	A*	No	(A)	Yes
Trigger factors – ability to avoid / manage migraine trigger factors	(B)	Yes		No
Section 3: Treatment effectiveness and financial impact				
Satisfaction with treatment	A*		A*	
Confidence in treatment	A*		A*	
Consistency of treatment effect	A*		A*	
Medication use – the type (potency) and dose (how much) medication ***taken when experiencing*** a migraine or headache	A*		A*	
Medication use – the type (potency) and dose (how much) medication ***taken to prevent*** a migraine or headache	A*		A*	
Financial impact – the economic cost associated with migraine treatment (to the individual (out of pocket expenses)) and healthcare systems)	(A)		A*	
Use of healthcare resources in response to migraine	(A)		A*	
Section 4: Complications (Adverse Events)				
Treatment side effects – experiencing undesirable secondary effects from taking medications for migraine	A*		A**	

For peer review only

Mortality (death)	(A)		A**	
Included in Round 2 due to importance scores (A** or A*)	18		24	
Included in Round 2 due to qualitative feedback	9		7	
New outcomes added due to qualitative feedback	0		0	
**TOTAL number of outcomes for inclusion in Round 2**	**27**		**31**	

Footnote: Each outcome was assigned to one of six categories reflecting levels of agreement: outcomes classified A** and A* would be included in round 2.

- A** if in both sub-panel groups the median rating is 9
- A* if in both sub-panel groups ≥70% rate an outcome ≥7
- (A) if in both sub-panel groups the median outcome rating is ≥7
- (B) if the median rating for an outcome is ≥7 in only one sub-panel group

For peer review only

Round two

Round two questionnaires contained 27 (episodic) and 31 (chronic) domains. Round two was completed by 23/33 (70%) and 29/31 (93%) health professionals and 33/42 (79%) and 25/34 (74%) patients for chronic and episodic migraine, respectively (totalling 54 episodic (83%) and 56 chronic (75%) migraine questionnaires completed).

When prioritised according to the top 10 and top 50% of domains, several overriding 'meaningful' domains could be described (Tables 2 a-b); six of which were common to both episodic and chronic migraine: pain, usual activities, cognition, adverse events, overall health, associated symptoms. Respondents to the episodic migraine questionnaire also prioritised self-management, whilst medication use was prioritised by chronic migraine respondents.

Table 2a. Delphi Round 2. Results of domain prioritisation for Episodic Migraine (combined panels n=27)**

Rank*	**Proposed 'merged' domain and definition**	**Top 10/27 prioritised domains**	**Top 50% of prioritised domains (rank 1 to 13/27 inclusive)**	**Lower 50% of prioritised domains (rank 14 to 27 inclusive)**
**1**	**Pain** - ***Experience of an unpleasant sensation that aches or hurts in the head; the frequency, severity and duration of this pain is important***	Pain associated with Migraine – experience of an unpleasant sensation that aches or hurts (1/27)		
		Frequency of pain associated with a migraine (2/27)		
		Severity or intensity of pain associated with a migraine (3/27)		
		Duration of pain associated with a migraine (4/27)		
**2**	**Usual activities** - ***Being able to carry out usual activities (including paid or unpaid work, study, domestic chores, care or support for family or close friends) to an acceptable or usual***	Being able to carry out ***activities related to work*** (paid or unpaid) ***or study*** to an acceptable or usual standard (5/27)		
			Family roles – able to provide usual care or support for family or close friends, including ability to commit activities (11/27)	

For peer review only - http://bmjopen.bmj.com/site/about/guidelines.xhtml

	standard - ***Being able to participate in, or commit to, usual activities***		Needing to take time-off work (paid or unpaid) or study (13/27)	
				Participation in social or leisure activities – ability to participate in, or commit to, social or leisure activities (22/27)
**3**	**Cognition** - ***Difficulty concentrating, ability to 'think clearly', or to remember things***	Cognitive function – difficulty concentrating, ability to think 'clearly' or to remember things (6/27)		
**4**	**Adverse events**	Treatment side-effects – experiencing undesired secondary effects from taking medications for migraine (7/27)		
**5**	**Overall health**	An individual's general health status; the ability to 'live a normal life' (8/27)		
**6**	**Self-management**	Trigger factors – the ability to avoid / manage migraine trigger factors (9/27)		
			Self-management – ability to effectively decrease / minimise/ control the impact of migraine on oneself (e.g. pharmaceutical, diet, lifestyle choices etc) (11/27)	
				Unpredictability of a migraine – uncertainty of being symptom free or able to engage in activities (17/27) ***** prioritised in top 10 (10/27) by patients***
**7**	**Associated symptoms**	Increased sensitivities – to light, sound, smell or touch (10/27)		
				Vomiting and/ or feelings of nausea (15/27) **** prioritised in top 10 (8/27) by HCPs**
				Physical fatigue – experiencing physical fatigue, tiredness, lacking in energy, feeling physically exhausted (18/27) ***** prioritised in top 50% (11/27) by patients***
8	Medication use			Satisfaction with treatment (14/27) ***** prioritised in top 10 (9/27) by HCPs***
				The type (potency) and dose (how much) of a medication ***taken when experiencing*** a migraine (16/27)

For peer review only - http://bmjopen.bmj.com/site/about/guidelines.xhtml

				** ***prioritised in top 50% (11/27) by HCPs***
				The type (potency) and dose (how much) of a medication ***taken to prevent*** a migraine (21/27)
				Consistency in treatment (23/27)
				Confidence in treatment (25/27)
9	Emotional well-being			Anxiety (19/27)
				Depression (19/27) ** ***prioritised in top 50% (13/27) by patients***
				Stress (24/27)
				Self-worth (24/27)
				Isolation (27/27)

Footnote:
*Top 7 grouped domains – informed by top 10 and top 50% of prioritised domains (13/27).
** 6 domains prioritised differently between the two panels; considered further in Round 3.

Table 2b. Delphi Round 2. Results of domain prioritisation for Chronic Migraine (combined panels n=31) **.

Rank*	**Domain and definition**	**Top 10/31 prioritised domains**	**Top 50% of prioritised domains (rank 1 to 15/31 inclusive)**	**Lower 50% of prioritised domains (rank 16 to 31 inclusive)**
**1**	**Pain**<br>- *Experience of an unpleasant sensation that aches or hurts in the head; the frequency, severity and duration of this pain is important*	Severity or intensity of pain associated with a migraine (1/31)		
		Pain associated with Migraine – experience of an unpleasant sensation that aches or hurts (2/31)		
		Frequency of pain associated with a migraine (3/31)		
		Duration of pain associated with a migraine (4/31)		
**2**	**Usual activities**<br>- *Being able to carry out usual activities (including paid or unpaid work, study, domestic chores, care or support for family or close friends) to an acceptable or usual standard*<br>- *Being able to participate in, or commit to, usual activities*	Being able to carry out usual tasks or daily activities inside or outside the home (not related to paid employment) *that support an independent lifestyle* – such as tidying one's home, walking short distances, managing finance, driving, usual technology *(instrumental activities of daily life)* (5/31)		
		Being able to carry out activities related to work (paid or unpaid) or study to an acceptable or usual standard (6/31)		
			Needing to take time-off work (paid or unpaid) or study (11/31)	
				Family roles – able to provide usual care or support for family or close friends, including ability to commit activities (19/31)

For peer review only - http://bmjopen.bmj.com/site/about/guidelines.xhtml

				Participation in social or leisure activities – ability to participate in, or commit to, social or leisure activities (22/31)
**3**	**Cognition**<br>- *Difficulty concentrating, ability to 'think clearly', or to remember things*	Cognitive function – difficulty concentrating, ability to think 'clearly' or to remember things (7/27)		
**4**	**Adverse events**	Treatment side-effects – experiencing undesired secondary effects from taking medications for migraine (8/31)		
				Mortality (death) (26/31)<br>***** prioritised in top 50% (15/31) by HCPs***
**5**	**Associated symptoms**	Increased sensitivities – to light, sound, smell or touch (9/31)		
		Physical fatigue – experiencing physical fatigue, tiredness, lacking in energy, feeling physically exhausted (10/31)		
			Sleep quality – being able to have a restful sleep (14/31)	
			Needing to rest or lie down because of a headache (15/31)	
**6**	**Medication use**		Satisfaction with treatment (12/31)	
				The type (potency) and dose (how much) of a medication taken to prevent a migraine (21/31)
				Consistency in treatment effect (23/31)
				The type (potency) and dose (how much) of a medication taken during a migraine (24/31)
				Confidence in treatment (28/31)
**7**	**Overall health**		An individual's general health status; the ability to 'live a normal life' (13/31)	
8	Emotional well-being			Stress – feelings of distress, frustration or irritation (16/31)<br>***** prioritised in top 10 (10/31) by HCPs***
				Anxiety – concerned, worried, fearful or anxious (20/31)
				Self-worth – feeling like a burden to others; can include feeling valued or helpless; accepted or rejected; feelings of self-esteem (28/31)

For peer review only - http://bmjopen.bmj.com/site/about/guidelines.xhtml

				Feelings of isolation – feeling isolated; reduced social interactions (29/31)
				Social role – relationship with work colleagues or peers (31/31)
9	Self-management			Self-management – ability to effectively decrease / minimise/ control the impact of migraine on oneself (e.g. pharmaceutical, diet, lifestyle choices etc) (17/31)
				Unpredictability of a migraine – uncertainty of being symptom free or able to engage in activities (18/31) ***** prioritised in top 50% (14/31) by patients***
10	Financial impact			Economic cost associated with treatment for headache (to the individual (out-of-pocket expenses) and healthcare system) (25/31)
				Use of healthcare resources in response to headache (30/31)

Footnote:
* Top 5 grouped domains – informed by top 10 prioritised domains. Top 7 grouped domains – informed by top 13 and top 50% of prioritised domains (15/31).
** 3 domains prioritised differently between the two panels; considered further in Round 3.

Group discrepancies for both episodic and chronic migraine included patients' prioritisation of overall health, physical fatigue, unpredictability and self-management. People with episodic migraine also prioritised emotional wellbeing. Although awarded fewer points, people with chronic migraine prioritised the importance of social role and emotional wellbeing. In contrast, healthcare professionals prioritised treatment satisfaction, treatment side-effects and vomiting/ nausea for episodic migraine, and mortality and stress for chronic migraine.

Round three

Round three was completed by 23/23 (100%) and 21/29 (72%) health professionals, and 29/33 (88%) and 23/25 (92%) patients for chronic and episodic migraine, respectively (totalling 52/56 (93%) for chronic migraine and 44/54 EM (81%) for episodic migraine. Six and three domain discrepancies (top 10 or top 50% for one group only) were considered for episodic migraine (treatment satisfaction; vomiting/ feelings of nausea; medication taken during a migraine; unpredictability; physical fatigue; depressive mood) and chronic migraine (stress; mortality; unpredictability), respectively (Appendix Table 3).

For peer review only - http://bmjopen.bmj.com/site/about/guidelines.xhtml

The seven domains for episodic migraine were retained (>76% across sub-panels; >84% combined) (Table 3) and a new domain 'Treatment Satisfaction' proposed (>70% healthcare professionals; 68% combined) (Appendix Table 3).

Table 3. Delphi Round 3: results of voting for domains for episodic and chronic migraine

Proposed CORE DOMAINS for EM and CM *(For voting in Round 3)*			EPISODIC MIGRAINE *Voting*			CHRONIC MIGRAINE *Voting*		
Prioritised domains *(informed by Round 2)*	Proposed 'Meaningful Domain' and definition *(bold text informed by R3 qualitative feedback)*	Q	Patient (n=23)	HCPs (n=21)	Combined (n=44)	Patient (n=29)	HCPs (n=23)	Combined (n=52)
• Pain associated with migraine – an unpleasant sensation that aches or hurts<br>• Frequency of pain associated with migraine<br>• Severity or intensity of pain associated with migraine<br>• Duration of pain associated with migraine	***PAIN***<br>- Experience of an unpleasant sensation in the head that aches or hurts and is associated with experiencing a migraine;<br>- the components of frequency, severity and duration of pain are all important<br><br>*Qualitative feedback supported the addition of:*<br>- *unpleasant sensation in the head …* ***face, neck and/or shoulders …***	a.<br>b.	100.0%<br>82.6%	100.0%<br>100.0%	100.0%<br>90.9%	96.6%<br>89.7%	86.9%<br>95.7%	92.3%<br>92.3%
• An individual's health status; the ability to live a 'normal' life	**OVERALL HEALTH**<br>- An individual's health status; the ability to live a 'normal' life<br><br>*Qualitative feedback challenged the concept or 'normal life' and the lack of clarity re a focus on* ***migraine-specific or general quality of life.*** *To be explored during the consensus meeting.*	a.<br>b.	100.0%<br>87.0%	90.5%<br>81.0%	95.5%<br>84.1%	96.6%<br>89.7%	87.0%<br>78.3%	92.3%<br>84.6%
• Being able to carry out *activities related to work (paid or unpaid) or study* to an acceptable or usual standard<br>• Family roles-able to provide usual care or support for family or close friends,	**USUAL ACTIVITIES**<br>- Being able to carry out usual activities (including paid or unpaid work, study, domestic chores, family or leisure activities, care or support for family or close friends) to an acceptable or usual standard	a.<br>b.	95.7%<br>95.7%	81.0%<br>76.2%	88.6%<br>86.4%	100.0%<br>89.7%	95.7%<br>95.7%	98.1%<br>92.3%

For peer review only - http://bmjopen.bmj.com/site/about/guidelines.xhtml

including to commit to activities *(EM only)*<br>• Need to take time-off work (paid or unpaid) or study<br>• Being able to carry out usual tasks or daily activities inside or outside the home (not related to employment) *that support an independent lifestyle* – such as tidying one's home, walking short distances, managing finance, driving, using technology *(CM only)*	- Being able to participate in or commit to usual activities<br><br>*Qualitative feedback supported the importance of including 'unpredictability' in the definition:*<br>- ***Being able to plan, commit to, or participate in usual activities, including work, usual social or caring roles (due to the unpredictability of a migraine)***							
• Cognitive function – difficulty concentrating, ability to think 'clearly' or to remember things	**COGNITIVE FUNCTION**<br>- Difficulty with concentrating, thinking clearly, or remembering things;<br><br>*Qualitative feedback supported the addition of:*<br>- ***difficulty with communication (word finding, slow or slurred speech)***	a.<br>b.	95.7%<br>91.3%	100.0%<br>90.5%	97.7%<br>90.9%	96.6%<br>93.1%	95.7%<br>95.7%	96.1%<br>94.2%
• Treatment side-effects – experiencing undesired secondary effects from taking medications for migraine	**ADVERSE EFFECTS**<br>- Experiencing undesired secondary effects from taking medications for migraine<br><br>*Qualitative feedback supported adoption of the CTCAE standardised definition of adverse events:*<br>- ***'any unfavourable and unintended sign, symptom, or disease temporarily associated with the use of a medical treatment or procedure that may or may not be considered related to the medical***	a.<br>b.	100.0%<br>87.0%	100.0%<br>90.5%	100.0%<br>88.6%	89.7%<br>93.1%	95.7%<br>82.6%	92.3%<br>88.5%

	treatment or procedure.' (CTCAE ref)							
• Increased sensitivities – to light, sound, smell or touch<br>• Physical fatigue – experiencing physical fatigue, tiredness, lacking in energy, feeling physically exhausted *(CM ony)*<br>• Sleep quality – being able to have a restful sleep *(CM only)*<br>• Needing to rest or lie down because of a headache *(CM only)*	**ASSOCIATED SYMPTOMS**<br>- Increased sensitivities – to light **(photophobia)**, sound **(phonophobia)**, smell, touch, or movement<br>- Physical fatigue – experiencing physical fatigue, tiredness, lacking in energy, feeling physically exhausted (CM only)<br>- Sleep quality – being able to have a restful sleep (CM only)<br>- Needing to rest or lie down because of a headache (CM only)<br><br>*Qualitative feedback highlighted concern over the omission of the following components from associated symptoms:*<br>- *Visual disturbances.*<br>- *Depressive mood*<br>- *Vomiting / feelings of nausea*<br>*All to be explored in consensus meeting (for both EM and CM)*	a.<br>b.	87.0%<br>87.0%	100.00%<br>90.5%	93.2%<br>88.6%	96.6%<br>93.1%	73.9%<br>73.9%	86.5%<br>84.6%
• Satisfaction with treatment	**MEDICATION USE**<br><br>**Voting: Proposed domain REJECTED (values < 70%)**<br><br>Qualitative feedback highlighted the importance of a domain that was not just focused on medication use.<br><br>*NOTE: Voting on sub-group discrepancies (Table R3b) supported the inclusion of 'Treatment Satisfaction' as a domain within the EM domain set. Core group recommendation that 'TREATMENT SATISFACTION' is explored in consensus*	a.<br>b.	N/A	N/A	N/A	79.3%<br>72.4%	**69.6%**<br>**60.9%**	75.0%<br>**67.3%**

For peer review only

	meeting for both EM and CM							
• Trigger factors – the ability to avoid / manage migraine trigger factors<br>• Self-management – the ability to effectively decrease / minimise / control the impact of migraine on oneself (e.g. by pharmaceutical, diet, lifestyle choices etc.)	**SELF-MANAGEMENT**<br>- Ability to effectively decrease / minimise / control the impact of migraine on oneself (e.g. by pharmaceutical, diet, lifestyle choices etc.)<br>- Ability to avoid / manage migraine trigger factors<br><br>*Qualitative feedback – proposed a more positive definition:*<br>- ***Living better with migraine through lifestyle, dietary, pharmaceutical choices and taking an active part in long-term management of migraine with education and support.***<br>- ***Enabling patients to become active partners in their migraine treatment***	a.<br><br>b.	95.7%<br><br>91.3%	85.7%<br><br>81.0%	90.9%<br><br>86.4%	N/A	N/A	N/A

Footnote:

Participants were invited to vote (Yes/No): a. Are you happy with the grouping of prioritised domains (Yes/No)? ; b. Are you happy with the proposed 'meaningful' domain and definition (Yes/No)?

N/A: Not applicable. Panellists did not vote in this domain.

For peer review only

Voting on sub-group discrepancies further supported the inclusion of vomiting/feelings of nausea, physical fatigue and depressive mood within the developing core domain set for episodic migraine (Appendix Table 3). Qualitative feedback in the questionnaire supported a more positive re-phrasing of the concept of self-management.

Six of the seven domains for chronic migraine were retained (>73% across sub-panels; >80% combined) (Table 4).

For peer review only - http://bmjopen.bmj.com/site/about/guidelines.xhtml

Table 4. Delphi Round 3: results of voting on sub-panel discrepancies.

Outcome to be voted on (R3)			EPISODIC MIGRAINE *Voting*			CHRONIC MIGRAINE *Voting*		
Discrepancies (outcomes rated in top 50% by one sub-panel)	Proposed Domain and definition	Q	Patient (n=23)	HCPs (n=21)	Combined (n=44)	Patient (n=29)	HCPs (n=23)	Combined (n=52)
Ranked highly by healthcare professionals (HCPs)								
• *HCP 9/27; Patients 20/27 (EM)*	• **Satisfaction with Treatment**	a.	65.2%	**71.4%**	68.2%	-	-	-
• *HCP 8/27; Patients 25/27 (EM)*	• **Vomiting and/ feelings of nausea**	a.	60.9%	**71.4%**	65.9%	-	-	-
• *HCP 12/27; Patients 18/27 (EM)*	• Type (potency) and dose (how much) of a medication when experiencing a migraine	a.				-	-	-
• *HCP 10/31; Patients 20/31 (CM)*	• Stress – feelings of distress, frustration or irritation	a.	-	-	-	58.6%	47.8%	53.9%
• *HCP 15/31; Patients 29/31 (CM)*	• Mortality (death)	a.	-	-	-	20.7%	17.4%	19.2%
Ranked highly by patients								
• *Patients 10/27; HCPs 21/27 (EM)* • *Patients 14/31; HCPs 31/31 (CM)*	• **Unpredictability of a migraine – uncertainty of being symptom-free or able to engage in activities**	a.	**82.6%**	61.9%	**72.7%**	**96.6%**	**69.6%**	**84.6%**
• *Patients 11/27; HCPs 23/27 (EM)*	• **Physical fatigue – experiencing physical fatigue, tiredness, lacking in energy, feeling physically exhausted**	a.	**69.6%**	52.4%	61.4%	-	-	-
• *Patients 10/27; HCPs 21/27 (EM)*	• **Depressive mood – feeling sad, feeling down, feeling sorry for oneself, or feeling depressed**	a.	**69.6%**	42.9%	56.8%	-	-	-

Footnote: Panellists were asked to indicate (Yes/No): a. Should the following outcomes be included in a core set for studies of EM / CM (respectively)?

'Medication Use' was rejected (<70%), and a redefining as 'Treatment Satisfaction' proposed. Qualitative feedback also highlighted the omission of 'visual disturbances' from 'Associated Symptoms', and the movement of 'Sleep Quality' to 'Usual Activities'.

For peer review only - http://bmjopen.bmj.com/site/about/guidelines.xhtml

For both episodic and chronic migraine, qualitative feedback highlighted the importance of communication difficulties within cognitive function; further consideration of vomiting/nausea, fatigue and depressive mood as additional ‘Associated Symptoms’; and unpredictability and ability to uphold usual commitments within ‘Usual Activities’. Further clarification of the concept of ‘Overall Health’ – for example, general or migraine-specific health, was proposed and adoption of a standardised definition of ‘adverse events’ (Common Terminology Criteria for Adverse Events (CTCAE) [24].

The process defined seven core domains common to episodic and chronic migraine (Table 3). Additionally, episodic migraine included ‘self-management’.

Stage 2: Core Measurement Set

International expert panel face-to-face meeting

The one-day meeting took place at Warwick University in December 2018. Seven patients (three with episodic migraine and four with chronic migraine) and seven healthcare professionals/researchers (two doctors, two nurses, one physiotherapist, two measurement experts) participated from two countries (UK, Portugal). Ten core group members, including two patient research partners (GP, BB), attended.

Pain – was re-defined as migraine-specific pain and endorsed as an inner core domain for episodic and chronic migraine (>70%) (Table 5; Figure 2). Based on review of existing measures and group discussion voting supported recommendation of the 11-point numerical rating scale (NRS) for assessing pain intensity [25] and number of headache/migraine days per month for pain frequency [1, 18]. Due to the complexities around the concepts of headache and migraine, it was recommended that the specific terminologies should be defined by individual studies.

Overall health – was re-defined as ‘migraine-specific quality of life’ (MSQoL), endorsed as an inner core domain for both episodic and chronic migraine (Table 5; Figure 2). Presented with evidence for generic and migraine quality of life measures, participants preferred the Migraine Functional Impact Questionnaire (MFIQ) [2, 26]. The four domain scores of the MFIQ address several key concepts highlighted throughout the COSMIG process – including usual activities, physical, cognitive, social and emotional function. It also provides a global item score for usual activities.

Pain duration and ***associated symptoms*** were both judged as important, but not feasible for inclusion in all trials/research studies and thereby placed in the middle circle (Table 5; Figure 2).

For peer review only - http://bmjopen.bmj.com/site/about/guidelines.xhtml

Self-management and ***Treatment satisfaction*** – were both considered important for both episodic and chronic migraine, but lack of conceptualisation and assessment supported their placement on the research agenda (outer circle) (Table 5; Figure 2).

Cognitive function and ***Usual activities*** were both rejected as independent core domains, but proposed as important components of migraine-specific quality of life (Table 5).

Adverse events – was rejected as a core domain, with the proposition that such reporting should be part of good clinical practice guidance (Table 5; Figure 2).

Table 5. Consensus meeting: results from small and large group discussions and voting.

Domain	Small group	Large group	Final decision [a]
**Pain**	***Domain***<br>Voting supported inclusion of Pain for EM and CM (>70%)<br>Three aspects of Pain included:<br>• intensity (11/11)<br>• frequency (10/11)<br>• duration (8/11)<br>Proposed domain refinement to 'Migraine-specific Pain'<br><br>***Measurement***<br>Voting for individual options did not exceed 70%<br>Preferred assessments:<br>Intensity: 11-point NRS (55%)<br>Frequency: Number of headache/migraine days (64%)<br>Duration: Cumulative hours per 28-days of moderate/severe pain (55%)	***Domain***<br>INNER core: Migraine-specific pain (no further voting required)<br><br>***Measurement***<br>Pain intensity: 11-point NRS (80%)<br><br>Pain frequency: Number of headache/migraine days (>70%)<br><br>Pain duration: No consensus. Proposed that daily capture (using paper or electronic diary) or retrospective capture using a questionnaire may not be feasible for all trials.<br>Voting: MIDDLE circle (89%)	***Domain – both EM and CM***<br>INNER core : Migraine-specific pain<br>Components: intensity and frequency<br><br>***Measurement***<br>Pain intensity – 11-point NRS (anchors 'no pain' and 'pain as bad as you can imagine')<br><br>Pain frequency<br>• number of headache / migraine days<br><br>Pain Duration: MIDDLE circle: important but not feasible for all trials / research studies
**Overall Health**	***Domain***<br>Voting supported redefining domain as Migraine-specific Quality of Life (73%)<br><br>***Measurement***<br>Migraine Functional Impact Questionnaire (72%)	***Domain***<br>INNER core: Migraine-specific Quality of Life (no further voting required)<br><br>***Measurement***<br>Migraine Functional Impact Questionnaire	***Domain – both EM and CM***<br>INNER core: Migraine-specific Quality of Life<br><br>***Measurement***<br>Migraine Functional Impact Questionnaire
**Adverse Events**	***Domain***<br>Voting supported the rejection of adverse events from the core domain set (82%)<br><br>***Measurement***<br>N/A	***Domain***<br>Recommendations were supported. Should be captured as part of good clinical practice guidance.	Not included in the COS for EM or CM
**Self-management**	***Domain***<br>No consensus on the inclusion (46%) / exclusion (54%) of self-management. Participants considered it to be important to both EM and CM, but requiring greater conceptualisation before it can be accurately measured	***Domain***<br>Group confirmed the importance of self-management for both EM and CM, but agreed that the lack of conceptualisation and method of assessment prevented inclusion in the COS.<br>Voting: RESEARCH AGENDA (73%)	***Domain and measurement – both EM and CM***<br>OUTER circle - Research Agenda: important but requiring further study
**Cognitive function**	***Domain***	***Domain***<br>Recommendations supported. The importance of cognitive	Not included as a separate core domain for EM or CM.

For peer review only - http://bmjopen.bmj.com/site/about/guidelines.xhtml

	Voting supported the rejection of cognitive function as a separate core domain (70%)<br><br>But participants supported cognitive function as an important concept.	function was supported and the potential for it to be captured with migraine-specific quality of life proposed.	Cognitive function is included within the new domain 'Migraine-specific Quality of Life' and will be assessed by the MFIQ
**Associated symptoms**	***Domain***<br>No consensus on the inclusion (50%) / exclusion (50%) of associated symptoms.<br><br>Participants discussed the importance of a wide range of associated symptoms – but capture of all would not be feasible in all trials (and hence not core)	***Domain***<br>Participants recognised pain as an important 'associated symptom' and the inclusion of several additional associated symptoms within the new domain 'MQoL' (captured by the MFIQ).<br><br>Capturing a larger number of associated symptoms, or specific additional symptoms - such as fatigue - should be study specific and not core.<br>Voting: MIDDLE circle (100%)	***Domain and measurement – both EM and CM***<br>MIDDLE circle: important but not feasible to include in all trials / research studies.
**Usual activities**	***Domain***<br>Voting supported the inclusion as a component of a new domain 'MQoL' (100%)<br><br>***Measurement***<br>Usual activities, as a component of MQoL to be assessed with the MFIQ (80%)	***Domain***<br>Recommendations were supported<br><br>***Measurement***<br>N/A	Not included as a separate core domain for EM or CM.<br><br>Usual activities is included within the new domain 'Migraine-specific Quality of Life' and will be assessed by the MFIQ
**Treatment satisfaction**	***Domain***<br>Considered important – but no consensus on the inclusion (64%) / exclusion(36%) of treatment satisfaction due to need for greater clarity	***Domain***<br>Group confirmed the importance of treatment satisfaction for both EM and CM, but agreed that the lack of conceptualisation and method of assessment prevented inclusion in the COS<br>Voting: RESEARCH AGENDA (100%)	***Domain and measurement – both EM and CM***<br>OUTER circle - Research Agenda: important but requiring further study

For peer review only

The result was a two domain Core Outcome Set for both EM and CM (COSMIG) (Table 5; Figure 2):

1) Migraine-specific pain: intensity assessed with the 11-point NRS and frequency as the number of headache/migraine days over a specified period; and

2) Migraine-specific quality of life – assessed with the MFIQ [26].

Discussion

The COSMIG process has identified two core domains - pain and migraine-specific quality of life – that are recommended as part of *a priori*-designated outcomes in future preventive intervention clinical trials for both episodic and chronic migraine. Pain assessment should include both intensity, measured

For peer review only - http://bmjopen.bmj.com/site/about/guidelines.xhtml

with an 11-point NRS, and frequency, assessed as the number of headache/migraine days per 28 days. Migraine-specific quality of life should be assessed with the Migraine Functional Impact Questionnaire (MFIQ) [26]. Complex concepts around headache and migraine meant the group were not able to make recommendations for the phrasing of questions on pain severity (e.g., worst, average or typical) or the definition of a migraine/headache day. Thus the specific terminologies should be defined, and reported, by the needs of individual studies. Likewise the specific timing of assessments should be driven by the requirements of the study.

The group preferred the MFIQ over other measures of migraine related quality of life such as the Migraine Specific Quality-of-Life Questionnaire MSQv2.1 because participants, in particular patient participants, felt its domains best reflected the impact migraine has on people's lives. This matches the aims of the original developers who specifically sought to address gaps in existing patient reported outcomes [27]. A licence is needed to use the MFIQ available from Legal@evidera.com. The owners advise us that it will be available free of charge for non-commercial research (email Evidera 15 May 2020, personal communication).

Pain duration and associated symptoms are important, but are not considered core. How to assess self-management and treatment satisfaction requires further research before recommendations can be made.

The COSMIG recommendations contrast with previous guidance for trials of prophylaxis in chronic migraine that recommend a single primary outcome derived from headache/migraine days. Patient-reported headache-related quality of life appears last in order of the secondary outcomes [1] and guidelines for trials of prophylaxis in episodic migraine do not include quality of life as an outcome [13].

Informed by current good practice guidance in core outcome set development [9, 14], this study included international participation from patient and professional groups in an on-line Delphi study and a subsequent face-to-face meeting. Whilst individuals from 14 countries were included in the Delphi study, participants from just two countries (England and Portugal) contributed to the face-to-face meeting. However, wide international involvement throughout the Delphi study improved international reach and helps ensure relevance of the recommendations.

Active pre-engagement with potential participants in the Delphi study enabled targeted follow-up of non-responders in round one [28]. The high response rates – approximating 80% - reported for patient participants, contrasted with the 50% response rates for healthcare professionals completing round one. This may reflect that the healthcare professionals were asked to complete two questionnaires (episodic and chronic migraine), whilst patients completed just one. Subsequent response rates for

For peer review only - http://bmjopen.bmj.com/site/about/guidelines.xhtml

rounds two and three were high, with response rates from both sub-panels exceeding 70%, paralleling the reduction in length of the questionnaire in both rounds.

We relied on participant self-identification of diagnosis of episodic/chronic migraine. Any misclassification is unlikely to have any substantive impact on our findings. The study included a broad age-range of patient participants. Similarly, the healthcare professionals involved had a broad spectrum of experience in the care of patients with migraine and in migraine-related research.

Working collaboratively with patient research partners throughout the research contributed to the crafting of 'meaningful' domains at each stage of the Delphi process, giving validity to the proposed lists [17]. The initial Delphi questionnaire provided a comprehensive reflection of domains that might be assessed in chronic or episodic migraine. Additional domains were not proposed by participants in round one, supporting the comprehensiveness and relevance of content. Patient partners checked the comprehensibility and relevance of short-listed methods of assessment presented to participants in the consensus meeting, contributing to the debate and supporting lay participants during group discussions. All patient partners contributed to manuscript edits throughout the write-up phase.

The recommended COSMIG core set should be complemented by additional trial outcomes pertinent to the particular intervention being evaluated. However, standardisation of core data collection is strongly advised to reduce the potential for systematic bias and enhance the quality of patient-reported outcomes data [8, 9]. The remaining discrepancies between patients and healthcare professionals are important. Patients attached greater importance to fatigue, unpredictability, emotional impact, cognitive function and self-management and lower prioritisation on concerns around vomiting/nausea than did professionals. More work is needed on how to evaluate the self-management and treatment satisfaction domains.

Through an international collaboration between patients, researchers and health professionals, we have facilitated consensus on a Core Outcome Set for reporting on preventative intervention trials and research studies in adults with episodic or chronic MIGraine (COSMIG). We recommend that both pain (intensity and frequency) and migraine-specific quality of life are included as core domains. To support meaningful comparisons across studies, we recommend that pain intensity be assessed with a NRS [25] and frequency by determining the number of migraine days; migraine-specific quality of life should be assessed with the MFIQ [26]. The timing of assessments should be determined by individual studies.

For peer review only - http://bmjopen.bmj.com/site/about/guidelines.xhtml

Author Affiliations

[1] Warwick Research in Nursing, Warwick Medical School, University of Warwick. Coventry CV4 7AL.

[2] Warwick Clinical Trials Unit, Warwick Medical School, University of Warwick. Coventry CV4 7AL.

[3] Institute of Health Sciences, Kristiana University College, Norway.

[4] School of Psychological, Social and Behavioural Sciences, Coventry University.

[5] Department of Neurology, Albert Einstein College of Medicine, USA.

[6] Nuffield Department of Primary Care, Oxford University, Oxford.

[7] Outcomes Insight Consulting, QualityMetric Inc.

[8] Neurology Department, St George's University Hospitals NHS Foundation Trust.

[9] The Headache Group, National Hospital for Neurology and Neurosurgery, University College London Hospitals NHS Foundation.

Acknowledgements

We are very grateful to members of the International Steering Group (RL, RJ) for their oversight of the study. We are very grateful to everyone who participate in the Delphi study and attended the consensus meeting.

Competing interests

MU and RF are directors and shareholders of Clinvivo Ltd. MU recused himself from any discussions related to the choice of Delphi platform for this study. MU is chief investigator or co-investigator on multiple previous and current research grants from the UK National Institute for Health Research, Arthritis Research UK and is a co-investigator on grants funded by the Australian NHMRC. He is an NIHR Senior Investigator. He has received travel expenses for speaking at conferences from the professional organisations hosting the conferences. MU and RF are part of an academic partnership with Serco Ltd related to return to work initiatives. MU is a co-investigator on two NIHR funded studies receiving additional support from Stryker Ltd. He has accepted honoraria for teaching/lecturing from CARTA. He was an editor of the NIHR journal series, and a member of the NIHR Journal Editors Group, for which he received a fee.

For peer review only - http://bmjopen.bmj.com/site/about/guidelines.xhtml

For peer review only

MSM serves on the advisory board for Abbott, Allergan, Eli Lilly, Medtronic, Novartis, TEVA; has received payment for the development of educational presentations from Allergan, electroCore, Eli Lilly, Medtronic, Novartis, and TEVA; and, has received research grants from Abbott, electroCore and Medtronic

SP is a director of Health Psychology Services Ltd which, in part, provides psychological treatments for those with chronic pain.

No other competing interests declared.

Funding Statement

This study was funded by the National Institute for Health Research (NIHR) Programme Grants for Applied Research programme (RP-PG-1212-20018).The views expressed are those of the author(s) and not necessarily those of the NIHR or the Department of Health and Social Care.

Active collaboration with our patient research partners was supported by a 'Delivering Results Award' (IAS/23022/16) from the Institute of Advanced Studies, Warwick University.

Data sharing statement

De-identified data will be shard through the university accessible databases or repositories at Warwick University. Please contact Dr KH is additional information is required: email k.l.haywood@warwick.ac.uk

Authors' contribution

KH, MM, MU, RP, RF, RL, BD, SPe, SPa, VN, BB, LM and GP made substantial contributions to the conception and design of the study. KH, MM, BD, RF, RP, RL, SPe, BB, LM, GP and MU made substantial contributions to developing the protocol. KH, MM, MU, RP, RF, RL, RB, SPe, SPa, VN, KS, BB, LM and GP made substantial contributions to the acquisition of data, analysis and interpretation of data. All authors have been involved in drafting the manuscript or revising it critically for important intellectual content; given final approval of the version to be published.

For peer review only - http://bmjopen.bmj.com/site/about/guidelines.xhtml

References

1. Tassorelli C, Diener HC, Dodick DW, et al. Guidelines of the International Headache Society for controlled trials of preventive treatment of chronic migraine in adults. *Cephalalgia : an international journal of headache* 2018; 38: 815-832. 2018/03/06. DOI: 10.1177/0333102418758283.
2. Haywood KL, Mars TS, Potter R, et al. Assessing the impact of headaches and the outcomes of treatment: A systematic review of patient-reported outcome measures (PROMs). *Cephalalgia : an international journal of headache* 2018; 38: 1374-1386. 2017/09/19. DOI: 10.1177/0333102417731348.
3. Probyn K, Bowers H, Caldwell F, et al. Prognostic factors for chronic headache: A systematic review. *Neurology* 2017; 89: 291-301. 2017/06/16. DOI: 10.1212/wnl.0000000000004112.
4. Clarke M. Standardising outcomes for clinical trials and systematic reviews. *Trials* 2007; 8: 39. DOI: 10.1186/1745-6215-8-39.
5. Kirkham JJ, Gargon E, Clarke M, et al. Can a core outcome set improve the quality of systematic reviews?--a survey of the Co-ordinating Editors of Cochrane Review Groups. *Trials* 2013; 14: 21. 2013/01/24. DOI: 10.1186/1745-6215-14-21.
6. Jackson JL, Cogbill E, Santana-Davila R, et al. A Comparative Effectiveness Meta-Analysis of Drugs for the Prophylaxis of Migraine Headache. *PLoS One* 2015; 10: e0130733. 2015/07/15. DOI: 10.1371/journal.pone.0130733.
7. Silberstein SD, Lipton RB, Dodick DW, et al. Efficacy and safety of topiramate for the treatment of chronic migraine: a randomized, double-blind, placebo-controlled trial. *Headache* 2007; 47: 170-180. 2007/02/16. DOI: 10.1111/j.1526-4610.2006.00684.x.
8. Kirkham JJ, Dwan KM, Altman DG, et al. The impact of outcome reporting bias in randomised controlled trials on a cohort of systematic reviews. *BMJ (Clinical research ed)* 2010; 340: c365. 2010/02/17. DOI: 10.1136/bmj.c365.
9. Williamson PR, Altman DG, Bagley H, et al. The COMET Handbook: version 1.0. *Trials* 2017; 18: 280. 2017/07/07. DOI: 10.1186/s13063-017-1978-4.
10. Williamson PR, Altman DG, Blazeby JM, et al. Developing core outcome sets for clinical trials: issues to consider. *Trials* 2012; 13: 132. DOI: 10.1186/1745-6215-13-132.
11. Gorst SL, Gargon E, Clarke M, et al. Choosing Important Health Outcomes for Comparative Effectiveness Research: An Updated Review and User Survey. *PLOS ONE* 2016; 11: e0146444. DOI: 10.1371/journal.pone.0146444.
12. Tunis SR, Clarke M, Gorst SL, et al. Improving the relevance and consistency of outcomes in comparative effectiveness research. *Journal of comparative effectiveness research* 2016; 5: 193-205. 2016/03/02. DOI: 10.2217/cer-2015-0007.
13. Tfelt-Hansen P, Pascual J, Ramadan N, et al. Guidelines for controlled trials of drugs in migraine: third edition. A guide for investigators. *Cephalalgia : an international journal of headache* 2012; 32: 6-38. 2012/03/03. DOI: 10.1177/0333102411417901.
14. Boers M, Kirwan JR, Wells G, et al. Developing core outcome measurement sets for clinical trials: OMERACT filter 2.0. *Journal of clinical epidemiology* 2014; 67: 745-753. 2014/03/04. DOI: 10.1016/j.jclinepi.2013.11.013.
15. Ward F, Popay J, Porroche-Escudero A, et al. Mainstreaming public involvement in a complex research collaboration: A theory-informed evaluation. 2020; n/a. DOI: 10.1111/hex.13070.

For peer review only - http://bmjopen.bmj.com/site/about/guidelines.xhtml

16. Nichols VP, Ellard DR, Griffiths FE, et al. The lived experience of chronic headache: a systematic review and synthesis of the qualitative literature. 2017; 7: e019929. DOI: 10.1136/bmjopen-2017-019929 %J BMJ Open.
17. Orbai AM, de Wit M, Mease P, et al. International patient and physician consensus on a psoriatic arthritis core outcome set for clinical trials. *Annals of the rheumatic diseases* 2017; 76: 673-680. 2016/09/11. DOI: 10.1136/annrheumdis-2016-210242.
18. International Headache Society. ICHD / Guidelines, http://www.ihs-headache.org/ichd-guidelines (April 2020).
19. The National Institute of Neurological Disorders and Stroke (NINDS). Common Data Standards - Headache, https://www.commondataelements.ninds.nih.gov/headache (accessed 01/04/2020).
20. Dworkin RH, Turk DC, Farrar JT, et al. Core outcome measures for chronic pain clinical trials: IMMPACT recommendations. *Pain* 2005; 113: 9-19. 2004/12/29. DOI: 10.1016/j.pain.2004.09.012.
21. Turk DC, Dworkin RH, Allen RR, et al. Core outcome domains for chronic pain clinical trials: IMMPACT recommendations. *Pain* 2003; 106: 337-345. 2003/12/09. DOI: 10.1016/j.pain.2003.08.001.
22. IMMPACT. Initiative on Methods, Measurement, and Pain Assessment in Clinical Trials, http://www.immpact.org/ (accessed April 2020).
23. Tugwell P, Boers M, Brooks P, et al. OMERACT: an international initiative to improve outcome measurement in rheumatology. *Trials* 2007; 8: 38. 2007/11/28. DOI: 10.1186/1745-6215-8-38.
24. Institute NC. Common Terminology Criteria for Adverse Events (CTCAE), https://ctep.cancer.gov/protocoldevelopment/electronic_applications/ctc.htm (accessed April 2020).
25. Hjermstad MJ, Fayers PM, Haugen DF, et al. Studies comparing Numerical Rating Scales, Verbal Rating Scales, and Visual Analogue Scales for assessment of pain intensity in adults: a systematic literature review. *Journal of pain and symptom management* 2011; 41: 1073-1093. 2011/05/31. DOI: 10.1016/j.jpainsymman.2010.08.016.
26. Hareendran A, Skalicky A, Mannix S, et al. Development of a New Tool for Evaluating the Benefit of Preventive Treatments for Migraine on Functional Outcomes – The Migraine Functional Impact Questionnaire (MFIQ). 2018; 58: 1612-1628. DOI: 10.1111/head.13420.
27. Kawata AK, Hareendran A, Shaffer S, et al. Evaluating the Psychometric Properties of the Migraine Functional Impact Questionnaire (MFIQ). *Headache* 2019; 59: 1253-1269. 2019/06/07. DOI: 10.1111/head.13569.
28. Gargon E, Gorst SL and Williamson PR. Choosing important health outcomes for comparative effectiveness research: 5th annual update to a systematic review of core outcome sets for research. *PloS one* 2019; 14: e0225980-e0225980. DOI: 10.1371/journal.pone.0225980.

For peer review only

For peer review only - http://bmjopen.bmj.com/site/about/guidelines.xhtml

Figure 1: Flow diagram outlining the development stages for the COSMIG

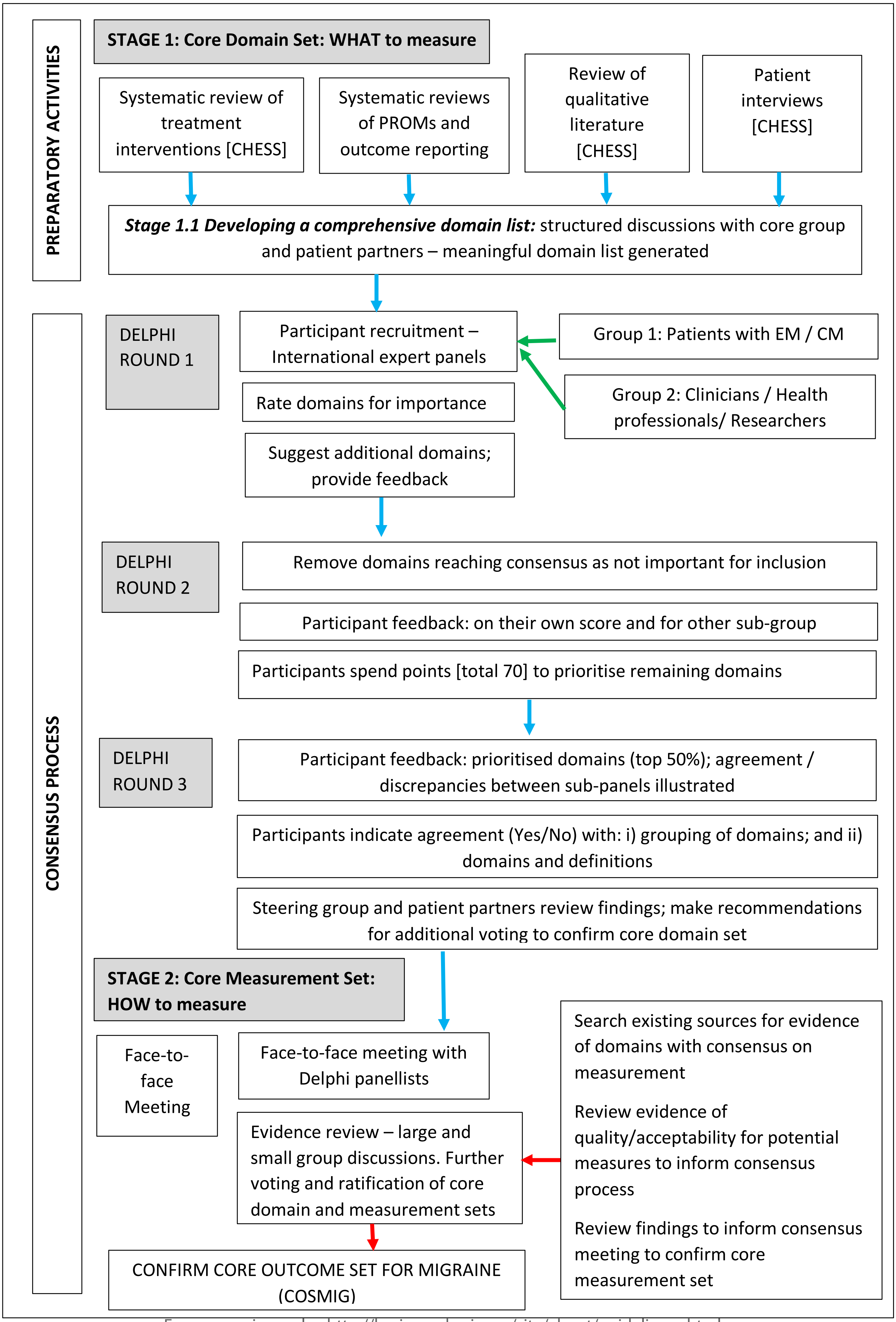

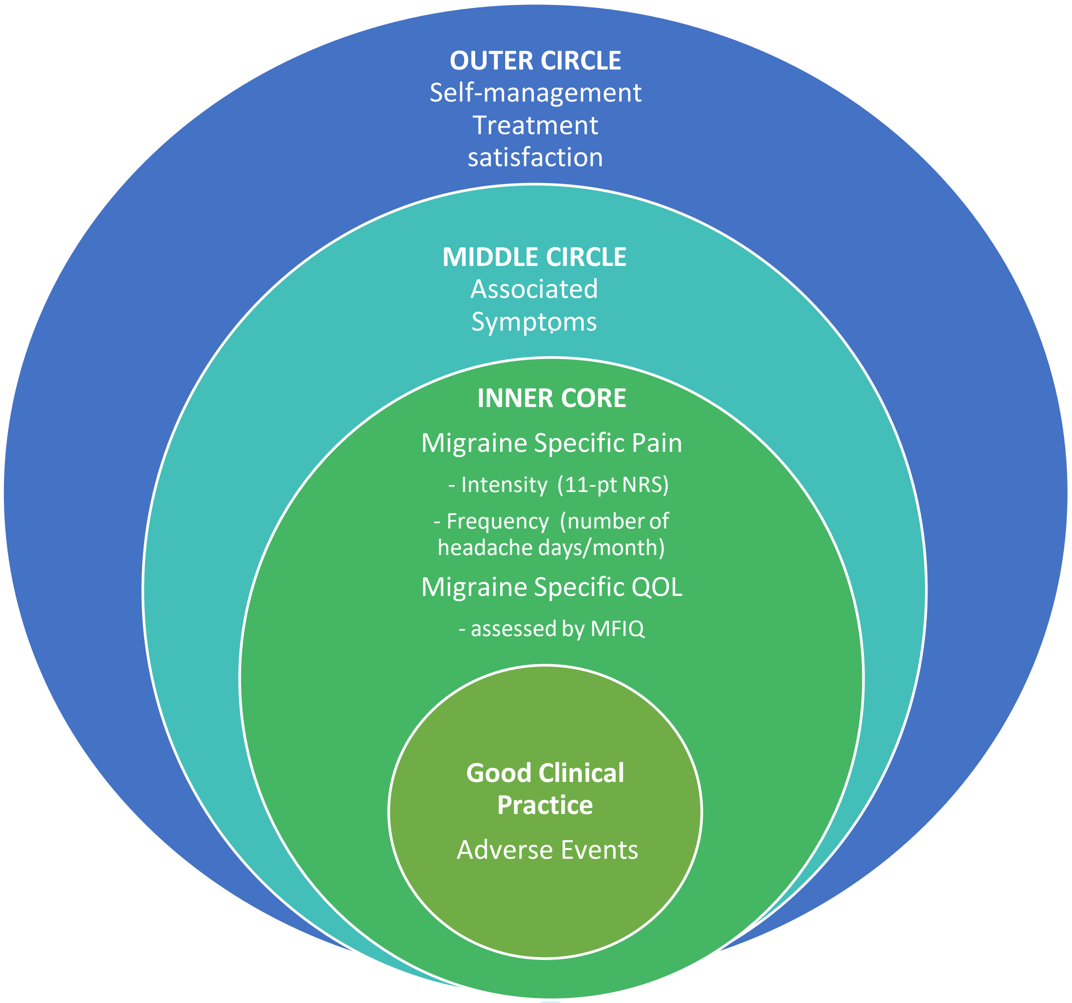

Figure 2. The Core Outcome Set for Episodic and Chronic Migraine (COSMIG):

Footnote: Core 'inner' circle: domain is unambiguous with an acceptable method of assessment; *Middle circle:* domain is important, but not feasible for all preventative trials and research studies; *Outer circle*: domain is important, but requires further study (research agenda).

For peer review only

APPENDIX

Appendix Table 1. Bespoke grading system to illustrate where consensus was achieved in the Delphi Round 1 for reviewed domains.

Grade	Level of agreement between groups	Decision rule
**A ****	If in both groups the median rating is 9	Include domain in Round 2
**A***	If in both groups ≥70% rate a domain ≥7	Include domain in Round 2
**A**	If in both groups the median domain rating is ≥7	Include domain in Round 2 if either group achieves a median score of 9 OR qualitative evidence supports further consideration
**B**	If the median rating for a domain is ≥7 in only one group	Include domain in Round 2 if either group achieves a median score of 9 OR qualitative evidence supports further consideration
**C**	If the median rating for the two groups combined is ≥4 and ≤6 and the median rating for no single group is ≤7	No progression to Round 2 (unless qualitative evidence supports further consideration)
**D**	If the median rating for the two groups combined is ≥1 and ≤3 and the median rating for no single group is ≤7	No progression to Round 2 (unless qualitative evidence supports further consideration)

Footnote: ‘both groups’ refers to – patient group and professionals group

For peer review only - http://bmjopen.bmj.com/site/about/guidelines.xhtml

Appendix Table 2. Background of professional participants in the Delphi process (Round 1).

	Chronic round	Episodic round
Clinician	6	5
Neurologist	13	12
Neurologist specialist interest headache	10	11
GP specialist interest headache	1	0
Nurse specialist	4	3
Chiro/osteopath/	2	1
Health Economist	2	1
Clinical Academic	8	9
Other health professional academic	2	0
Clinical Trialist	9	8
Systematic reviewer	6	5
Measurement expert	7	8

Footnote: participants could identify as having more than one background

For peer review only

For peer review only - http://bmjopen.bmj.com/site/about/guidelines.xhtml

Appendix Table 3. Delphi Round 3: results of voting on sub-panel discrepancies.

Outcome to be voted on (R3)			EPISODIC MIGRAINE *Voting*			CHRONIC MIGRAINE *Voting*		
Discrepancies (outcomes rated in top 50% by one sub-panel)	Proposed Domain and definition	Q	Patient (n=23)	HCPs (n=21)	Combined (n=44)	Patient (n=29)	HCPs (n=23)	Combined (n=52)
Ranked highly by healthcare professionals (HCPs)								
• *HCP 9/27; Patients 20/27 (EM)*	• **Satisfaction with Treatment**	a.	65.2%	**71.4%**	68.2%	-	-	-
• *HCP 8/27; Patients 25/27 (EM)*	• **Vomiting and/ feelings of nausea**	a.	60.9%	**71.4%**	65.9%	-	-	-
• *HCP 12/27; Patients 18/27 (EM)*	• Type (potency) and dose (how much) of a medication when experiencing a migraine	a.				-	-	-
• *HCP 10/31; Patients 20/31 (CM)*	• Stress – feelings of distress, frustration or irritation	a.	-	-	-	58.6%	47.8%	53.9%
• *HCP 15/31; Patients 29/31 (CM)*	• Mortality (death)	a.	-	-	-	20.7%	17.4%	19.2%
Ranked highly by patients								
• *Patients 10/27; HCPs 21/27 (EM)*<br>• *Patients 14/31; HCPs 31/31 (CM)*	• **Unpredictability of a migraine – uncertainty of being symptom-free or able to engage in activities**	a.	**82.6%**	61.9%	**72.7%**	**96.6%**	**69.6%**	**84.6%**
• *Patients 11/27; HCPs 23/27 (EM)*	• **Physical fatigue – experiencing physical fatigue, tiredness, lacking in energy, feeling physically exhausted**	a.	**69.6%**	52.4%	61.4%	-	-	-
• *Patients 10/27; HCPs 21/27 (EM)*	• **Depressive mood – feeling sad, feeling down, feeling sorry for oneself, or feeling depressed**	a.	**69.6%**	42.9%	56.8%	-	-	-

Footnote: Panellists were asked to indicate (Yes/No): a. Should the following outcomes be included in a core set for studies of EM / CM (respectively)?

For peer review only - http://bmjopen.bmj.com/site/about/guidelines.xhtml

Table 7. Consensus meeting: results from small and large group discussions and voting.

Domain	Small group	Large group	Final decision [a]
**Pain**	***Domain***<br>Voting supported inclusion of Pain for EM and CM (>70%)<br>Three aspects of Pain included:<br>• intensity (11/11)<br>• frequency (10/11)<br>• duration (8/11)<br>Proposed domain refinement to 'Migraine-specific Pain'<br><br>***Measurement***<br>Voting for individual options did not exceed 70%<br>Preferred assessments:<br>Intensity: 11-point NRS (55%)<br>Frequency: Number of headache/migraine days (64%)<br>Duration: Cumulative hours per 28-days of moderate/severe pain (55%)	***Domain***<br>INNER core: Migraine-specific pain (no further voting required)<br><br>***Measurement***<br>Pain intensity: 11-point NRS (80%)<br><br>Pain frequency: Number of headache/migraine days (>70%)<br><br>Pain duration: No consensus. Proposed that daily capture (using paper or electronic diary) or retrospective capture using a questionnaire may not be feasible for all trials.<br>Voting: MIDDLE circle (89%)	***Domain – both EM and CM***<br>INNER core : Migraine-specific pain<br>Components: intensity and frequency<br><br>***Measurement***<br>Pain intensity – 11-point NRS (anchors 'no pain' and 'pain as bad as you can imagine')<br><br>Pain frequency<br>• number of headache / migraine days<br><br>Pain Duration: MIDDLE circle: important but not feasible for all trials / research studies
**Overall Health**	***Domain***<br>Voting supported redefining domain as Migraine-specific Quality of Life (73%)<br><br>***Measurement***<br>Migraine Functional Impact Questionnaire (72%)	***Domain***<br>INNER core: Migraine-specific Quality of Life (no further voting required)<br><br>***Measurement***<br>Migraine Functional Impact Questionnaire	***Domain – both EM and CM***<br>INNER core: Migraine-specific Quality of Life<br><br>***Measurement***<br>Migraine Functional Impact Questionnaire
**Adverse Events**	***Domain***<br>Voting supported the rejection of adverse events from the core domain set (82%)<br><br>***Measurement***<br>N/A	***Domain***<br>Recommendations were supported. Should be captured as part of good clinical practice guidance.	Not included in the COS for EM or CM
**Self-management**	***Domain***<br>No consensus on the inclusion (46%) / exclusion (54%) of self-management. Participants considered it to be important to both EM and CM, but requiring greater conceptualisation before it can be accurately measured	***Domain***<br>Group confirmed the importance of self-management for both EM and CM, but agreed that the lack of conceptualisation and method of assessment prevented inclusion in the COS.<br>Voting: RESEARCH AGENDA (73%)	***Domain and measurement – both EM and CM***<br>OUTER circle - Research Agenda: important but requiring further study

For peer review only - http://bmjopen.bmj.com/site/about/guidelines.xhtml

**Cognitive function**	***Domain***<br>Voting supported the rejection of cognitive function as a separate core domain (70%)<br><br>But participants supported cognitive function as an important concept.	***Domain***<br>Recommendations supported. The importance of cognitive function was supported and the potential for it to be captured with migraine-specific quality of life proposed.	Not included as a separate core domain for EM or CM.<br><br>Cognitive function is included within the new domain 'Migraine-specific Quality of Life' and will be assessed by the MFIQ
**Associated symptoms**	***Domain***<br>No consensus on the inclusion (50%) / exclusion (50%) of associated symptoms.<br><br>Participants discussed the importance of a wide range of associated symptoms – but capture of all would not be feasible in all trials (and hence not core)	***Domain***<br>Participants recognised pain as an important 'associated symptom' and the inclusion of several additional associated symptoms within the new domain 'MQoL' (captured by the MFIQ).<br><br>Capturing a larger number of associated symptoms, or specific additional symptoms - such as fatigue - should be study specific and not core.<br>Voting: MIDDLE circle (100%)	***Domain and measurement – both EM and CM***<br>MIDDLE circle: important but not feasible to include in all trials / research studies.
**Usual activities**	***Domain***<br>Voting supported the inclusion as a component of a new domain 'MQoL' (100%)<br><br>***Measurement***<br>Usual activities, as a component of MQoL to be assessed with the MFIQ (80%)	***Domain***<br>Recommendations were supported<br><br>***Measurement***<br>N/A	Not included as a separate core domain for EM or CM.<br><br>Usual activities is included within the new domain 'Migraine-specific Quality of Life' and will be assessed by the MFIQ
**Treatment satisfaction**	***Domain***<br>Considered important – but no consensus on the inclusion (64%) / exclusion(36%) of treatment satisfaction due to need for greater clarity	***Domain***<br>Group confirmed the importance of treatment satisfaction for both EM and CM, but agreed that the lack of conceptualisation and method of assessment prevented inclusion in the COS<br>Voting: RESEARCH AGENDA (100%)	***Domain and measurement – both EM and CM***<br>OUTER circle - Research Agenda: important but requiring further study

Footnote: [a] *Core 'inner' circle:* domain is unambiguous with an acceptable method of assessment; *Middle circle:* domain is important, but not feasible for all preventative trials and research studies; *Outer circle*: domain is important, but requires further study (research agenda) – e.g. lacks conceptual clarity or method of assessment.

For peer review only - http://bmjopen.bmj.com/site/about/guidelines.xhtml

For peer review only

For peer review only

For peer review only - http://bmjopen.bmj.com/site/about/guidelines.xhtml

BMJ Open

A Core Outcome Set for Preventive Intervention Trials in Chronic and Episodic Migraine (COSMIG): An international, consensus-derived and multi-stakeholder initiative.

Journal:	*BMJ Open*
Manuscript ID	bmjopen-2020-043242.R1
Article Type:	Original research
Date Submitted by the Author:	04-May-2021
Complete List of Authors:	Haywood, Kirstie; University of Warwick Warwick Medical School, Warwick Research in Nursing<br>Potter, Rachel; University of Warwick Warwick Medical School, Clinical Trials Unit<br>Froud, Robert; Kristiania University College, Institute of Health Sciences<br>Pearce, Gemma; Coventry University Department of Psychology and Behavioural Sciences<br>Box, Barbara; University of Warwick Warwick Medical School, Clinical Trials Unit<br>Muldoon, Lynne; University of Warwick, Clinical Trials Unit, Warwick Medical School<br>Lipton, Richard; Albert Einstein College of Medicine Department of Neurology, Department of Neurology<br>Petrou, Stavros; Nuffield Department of Primary Care Health Sciences,<br>Rendas-Baum, Regina; QualityMetric, Outcomes Insight Consulting<br>Logan, Anne-Marie; St George's University Hospitals NHS Foundation Trust<br>Stewart, Kimberley; University of Warwick Warwick Medical School, Clinical Trials Unit<br>Underwood, Martin; University of Warwick Warwick Medical School, Clinical Trials Unit<br>Matharu, Manjit; University College London Hospitals NHS Foundation Trust National Hospital for Neurology and Neurosurgery, The Headache Group
**Primary Subject Heading**:	Neurology
Secondary Subject Heading:	Research methods
Keywords:	Migraine < NEUROLOGY, STATISTICS & RESEARCH METHODS, Clinical trials < THERAPEUTICS

SCHOLARONE™
Manuscripts

I, the Submitting Author has the right to grant and does grant on behalf of all authors of the Work (as defined in the below author licence), an exclusive licence and/or a non-exclusive licence for contributions from authors who are: i) UK Crown employees; ii) where BMJ has agreed a CC-BY licence shall apply, and/or iii) in accordance with the terms applicable for US Federal Government officers or employees acting as part of their official duties; on a worldwide, perpetual, irrevocable, royalty-free basis to BMJ Publishing Group Ltd ("BMJ") its licensees and where the relevant Journal is co-owned by BMJ to the co-owners of the Journal, to publish the Work in this journal and any other BMJ products and to exploit all rights, as set out in our licence.

The Submitting Author accepts and understands that any supply made under these terms is made by BMJ to the Submitting Author unless you are acting as an employee on behalf of your employer or a postgraduate student of an affiliated institution which is paying any applicable article publishing charge ("APC") for Open Access articles. Where the Submitting Author wishes to make the Work available on an Open Access basis (and intends to pay the relevant APC), the terms of reuse of such Open Access shall be governed by a Creative Commons licence – details of these licences and which Creative Commons licence will apply to this Work are set out in our licence referred to above.

Other than as permitted in any relevant BMJ Author's Self Archiving Policies, I confirm this Work has not been accepted for publication elsewhere, is not being considered for publication elsewhere and does not duplicate material already published. I confirm all authors consent to publication of this Work and authorise the granting of this licence.

For peer review only

A Core Outcome Set for Preventive Intervention Trials in Chronic and Episodic Migraine (COSMIG): An international, consensus-derived and multi-stakeholder initiative.

Haywood K[1], Potter R[2], Froud R[2,3], Pearce G[2,4], Box B[2], Muldoon L[2], Lipton RB[5], Petrou S[6], Rendas-Baum R[7], Logan A[8], Stewart K[2], Underwood M[2], Matharu M[9]; on behalf of the CHESS COSMIG group.

Author Affiliations

[1] Warwick Research in Nursing, Warwick Medical School, University of Warwick. Coventry CV4 7AL.

[2] Warwick Clinical Trials Unit, Warwick Medical School, University of Warwick. Coventry CV4 7AL.

[3] Institute of Health Sciences, Kristiana University College, Norway.

[4] School of Psychological, Social and Behavioural Sciences, Coventry University. CV1 5FB

[5] Department of Neurology, Albert Einstein College of Medicine and the Montefiore Headache Center, Bronx NY, USA.

[6] Nuffield Department of Primary Care, Oxford University, Oxford.

[7] Outcomes Insight Consulting, QualityMetric Inc.

[8] Neurology Department, St George's University Hospitals NHS Foundation Trust.

[9] The Headache and Facial Pain Group, Queen's Square Institute of Neurology and the National Hospital for Neurology and Neurosurgery, University College London Hospitals NHS Foundation. London. WC1N 3BG.

COSMIG Core Group:
Kirstie Haywood
Martin Underwood
Manjit Matharu
Brendan Davies
Rachel Potter
Stavros Petrou
Barbara Box
Gemma Pearce
Lynne Muldoon
Richard Lipton
Rigor Jensen
Vivien Nichols
Shilpa Patel
Kimberley Stewart

Corresponding author: Dr Rachel Potter r.potter@warwick.ac.uk

For peer review only

Main Text (4270)

Figure 1. Flow diagram outlining the development stages for the COSMIG
Figure 2. COSMIG: Core Outcome Set for Episodic and Chronic MIGraine.

Table 1. Delphi Round 1 Shortlisted domains
Table 2a. Delphi Round 2. Results of domain prioritisation for Episodic Migraine (combined panels)
Table 2b. Delphi Round 2. Results of domain prioritisation for Chronic Migraine (combined panels)
Table 3. Delphi Round 3. Results of voting for domains for episodic and chronic migraine
Table 4. Consensus meeting: results from small and large group discussions and voting.

Appendix

Appendix Table 1. Bespoke grading system to illustrate where consensus was achieved in the Delphi Round 1 for reviewed domains.
Appendix Table 2. Background of professional participants (expert panel) in the Delphi process (Round 1).
Appendix Table 3. Delphi Round 3. Results of voting on sub-panel discrepancies.

For peer review only

For peer review only - http://bmjopen.bmj.com/site/about/guidelines.xhtml

ABSTRACT (249/300 words)

Objective: Typically, migraine prevention trials focus on reducing migraine days. This narrow focus may not capture all that is important to people with migraine. Inconsistency in outcome selection across trials limits the potential for data pooling and evidence synthesis. In response, we describe the development of core outcome set for migraine (COSMIG).

Design: A two-stage approach sought to achieve international, multi-stakeholder consensus on both the core domain set (CDS) and core measurement set (CMS). Following construction of a comprehensive list of outcomes, expert panellists (patients, healthcare professionals and researchers) completed a three-round electronic-Delphi study to support a reduction and prioritisation of core domains and outcomes. Participants in a consensus meeting finalised the core domains and methods of assessment. All stages were overseen by an international core team, including patient research partners.

Results: There was good representation of patients (episodic (n=34) and chronic migraine (n=42)) and healthcare professionals (n=33) with high response and retention rates. The initial list of domains and outcomes was reduced from >50 to seven core domains for consideration in the consensus meeting, during which a two-domain core outcome set was agreed.

Conclusion: International and multi-stakeholder consensus emerged to describe a two-domain core outcome set for reporting research on preventive interventions for chronic and episodic migraine: migraine-specific pain and migraine-specific quality of life. Intensity of migraine-pain assessed with an 11-point numerical rating scale and the frequency as the number of headache/migraine days over a specified time-period. Migraine-specific quality of life assessed using the Migraine Functional Impact Questionnaire.

Strengths and limitations of this study:

- The research process and validity of results are strengthened by the co-collaboration with patient research partners throughout all stages of the research.
- A bespoke grading system to support the prioritisation of outcome domains between stakeholder groups (expert panels) is described.
- International, multi-stakeholder participation – patients, researchers, and a range of health professionals - in the on-line Delphi survey.
- Expert panel representation in the Delphi survey was largely from Europe and North America.
- The majority of participants in the face-to-face consensus meeting were from the UK.

For peer review only - http://bmjopen.bmj.com/site/about/guidelines.xhtml

BACKGROUND

International guidelines for the conduct of preventive studies for both episodic and chronic migraine specify that the primary outcome should be focussed on migraine days, or for chronic migraine on moderate to severe headache days [1]. Reviews of clinical trials of populations with chronic migraine and episodic migraine have identified substantial inconsistencies in outcomes reporting alongside often poorly defined outcomes [2, 3]. An important impact of these inconsistencies is to limit the potential for robust meta-analyses [4 5]. For example, a 2015 meta-analysis of drugs for the prophylaxis of migraine by Jackson et al [6] did not include data from the largest and most robust trial of topiramate for chronic migraine (n=307) that found a mean difference of 1.7 migraine/migrainous days per 28 days after 12 weeks [7]. The reviewers meta-analysed the data from two much smaller (n=32 & n=50), low quality studies, and reported an effect size of 8.4 headache days, the outcome specified for the meta-analyses, after 12 weeks. Data that cannot be interpreted or utilised can result in unacceptable and unethical research waste. There is also potential for selective outcomes reporting and associated reporting bias if consistent outcomes are not pre-specified [8, 9].

Improved consistency, accountability and transparency in outcome reporting can be achieved by using a Core Outcome Set (COS); a small, standardised group of outcomes that should be measured and reported, as a minimum, in all effectiveness trials for a specific health area [10-12].
Current international guidelines for conduct of prevention studies in episodic or chronic migraine have not developed outcome reporting recommendations in line with current best practice [1, 13]. Notably, patient input is markedly absent from these guidelines.
We describe here the development of a multiple-stakeholder, internationally endorsed, consensus-based COS applicable to preventative intervention trials and research studies in adults with episodic or chronic MIGraine (COSMIG).

Methods

Two key stages in core outcome set development are described (figure 1) [14]:

Stage 1) Defining the core domain set: WHAT to measure; i.e., the minimum number of health domains that should be assessed. A domain describes the concept or 'aspect of health or a health condition that needs to be measured to appropriately assess the effects of a health intervention' [14].

Stage 2) Recommending the core measurement set: HOW to measure, i.e., the minimum set of assessment methods that adequately correspond to the core domain set.

For peer review only - http://bmjopen.bmj.com/site/about/guidelines.xhtml

We prospectively registered COSMIG with the Core Outcomes Measures in Effectiveness Trials (COMET) initiative [http://www.comet-initiative.org/studies/details/953]. Ethical approval was gained from Warwick Medical School Biomedical and Scientific Research Ethics Committee REGO-2017-1921.

Patient and public involvement

Following good practice guidance [https://www.invo.org.uk/posttyperesource/before-you-start-involving-people/; [15] we worked collaboratively with our patient research partners, who all had experience of chronic or episodic migraine, throughout all stages of the research.

The COSMIG core group consisted of clinicians with expertise in headaches and migraine (MM,MU, BD), including two international members (RL,RJ), research scientists with expertise in clinical trials, Delphi technique, health measurement and qualitative research (MU,KH,RF,RP,SP,VN,SP,KS) and patient research partners (GP,BB,LM). Regular meetings were held between all group members to discuss the methodology for the Delphi study and the subsequent consensus meeting. The group met specifically between each Delphi round, to discuss results, confirm feedback and format for subsequent rounds.

Stage 1 Core Domain Set

Stage 1.1: Developing a comprehensive domain list

We first identified potential domains from systematic reviews [2, 3] and qualitative research [16]. Domains were written in plain English as on-line questionnaires: one questionnaire contained domains for episodic headache, and one for chronic headache. Questionnaires were piloted with the core team and researchers naïve to the study (n=12).

Stage 1.2: International modified-Delphi process

Our primary goal for our Delphi study was to refine and prioritise domains. The Delphi process seeks to establish consensus between a panel of experts following a structured process of questionnaire completion and systematic feedback.[17, 18] The panels are not intended to be representative of all headache specialists or people with migraine (as is the case when sampling from a definable population). We defined two expert panels external to the core research team: one comprised of expert patients with a target of up to 50 with chronic migraine (CM) and 50 with episodic migraine (EM); and a second panel (also up to 50) comprised of healthcare professionals and researchers, who were representative of their professions and well-placed to implement study findings[19].

For peer review only - http://bmjopen.bmj.com/site/about/guidelines.xhtml

Professionals included neurologists, nurse specialists, general practitioners, allied health professionals, researchers, and measurement experts. We sought consensus between experts on the core domain set.

Patients: We asked 13 national/international organisations to advertise the study on their social media platforms. Interested participants (≥18-years old) contacted the research team. We asked participants to self-diagnose/classify their migraines as episodic or chronic migraine. Patient participants completed episodic *or* chronic migraine questionnaires depending on their self-diagnosis.

Professionals: We invited national and international healthcare professionals (neurologists, GPs, nurses, psychologists, pharmacists, allied health professionals) and researchers (trialists, reviewers, health economists, measurement experts) involved in headache research identified through professional societies and from published research to participate. They were asked to complete *both* questionnaires.

The Delphi process had three sequential rounds with participants completing each prior round eligible to complete the next. The Delphi study administration and hosting of the on-line questionnaires was managed by Clinvivo Ltd.

Round 1 Participants rated the relative importance of each domain for inclusion in future research studies of chronic or episodic headache using a nine point numerical rating scale (range 1 to 3 'Not at all important', 4 to 6 'Uncertain', and 7 to 9 'Very important'). Participants could elaborate on their decisions by providing additional qualitative comment and/or provide additional domains for consideration and rating in subsequent rounds. Informed by an approach described by Orbai et al. (2017) [20], we devised a bespoke grading system to illustrate where consensus was achieved and to indicate more easily where participants in each panel disagreed in their judgement (Appendix Table 1). An *a priori* decision rule determined that only those outcome domains judged most favourably by one or both panels (patients and professionals) would be included in round two. That is, domains were included in round 2 if in both panels the median rating was 9 ('A**'), or if in both panels ≥70% rated a domain ≥7 ('A*'). If in both panels the median domain rating was is ≥7 ('A'), or the median rating for a domain was is ≥7 in just one panel ('B'), the domain could be included in round 2 if either panel achieved a median score of 9 or qualitative evidence supported further consideration.

Round 2 In round two we focused more specifically on migraine-specific (e.g. nausea and photophobia), rather than headache-specific, domains. Responses to round one were summarised and anonymous feedback provided. Participants all received their own score for each domain, and the group median scores. Further prioritisation was achieved by inviting participants to 'spend

For peer review only - http://bmjopen.bmj.com/site/about/guidelines.xhtml

points' (up to a maximum of 70) to illustrate how strongly they felt that a domain should be prioritised for inclusion in the core domain set; a maximum of 10 points could be allocated to any one outcome domain (11-point scale, 0 'Not a priority' to 10 'Absolute priority'). To ensure that sub-panel differences were considered, and any discrepancies highlighted, the results from both panels were considered both separately and combined: the top 10 and top 50% of prioritised domains were discussed between COSMIG core team members, informing the maintenance of, or, where the concepts of health were similar, grouping of domains into a single 'meaningful' domain.

Round 3 Responses to round two were summarised, highlighting the top 50% of prioritised domains and between-panel discrepancies. For those domains prioritised highly by just one panel (top 50%), participants were asked to reconsider if they should be included in the priority listing. If more than 70% of respondents selected 'yes', the domain was included. Finally, participants were asked to indicate by means of a dichotomous response if they: a) were happy with the grouping of prioritised domains; b) were happy with the proposed 'meaningful' domain and definition; and c) had additional comments. The frequency distribution of responses was calculated. Results from both sub-panels were again considered separately and combined.

Stage 2: Core Measurement Set

International expert panel face-to-face meeting

The purpose of the one-day meeting was to discuss the core domain set developed in our modified Delphi study, agree the core measurement set, and recommend the core outcome set. Importantly, participants were to consider that whilst a domain may be considered important, if an acceptable approach to measurement is not available, it is not appropriate to include the domain in a core outcome set.

We invited professionals from Europe and patients from the UK who had taken part in our Delphi study. Participants received an information pack with meeting objectives and domain/measurement information ahead of the meeting. Where existing consensus for potential measures was not available, the COSMIG core team reviewed key data sources for guidance and evidence of measurement quality, acceptability and feasibility for use in preventive studies of episodic or chronic migraine:[21]

- Migraine / headache:
    - Review of patient-reported outcome measures (PROMS)[2]
    - International Headache Society guidelines [1, 13, 22]

For peer review only - http://bmjopen.bmj.com/site/about/guidelines.xhtml

- National Institute for Neurological Disorders Common Data Elements – Headache (preventative treatment)[23]
- Chronic Pain and core outcome set development
  - Initiative on Methods, Measurement and Pain Assessment in Clinical Trials[24-26]
  - Outcome Measures in Rheumatology and Clinical Trials group [27]

The meeting started with an overview of the results of the Delphi process, prioritised domains, and the evidence-base underpinning potential methods of assessment. Participants were asked to consider three options when determining domain 'placement' within the final core outcome set [20]:

i) *Core 'inner' circle:* domain is unambiguous with an acceptable method of assessment;

ii) *Middle circle:* domain is important, but not feasible for all preventative trials and research studies;

iii) *Outer circle*: domain is important but requires further study (research agenda) – e.g. lacks conceptual clarity or method of assessment.

Semi-structured, small-group discussions with a mix of patients, healthcare professionals/ researchers and members of the core research team (including patient partners) ensued, covering each prioritised domain. Two facilitators each supported two rounds of discussion per domain. Outcome domains and methods of assessment were reviewed in terms of importance, quality, acceptability, and feasibility. Facilitators supported participant contribution and shared findings between groups to stimulate discussion. Following each small-group discussion, participants, with the exception of the core research team, were asked to indicate anonymously (paper-based questionnaire) their preference for domain inclusion (yes/no/don't know) and method of assessment (selecting one option from a short-list) in the core outcome set; an *a priori* definition of agreement required ≥ 70% of panellists to agree.

Next, small group discussions and results were presented to the whole group. Where there was agreement, no further discussion was required. Subsequent discussion focused on where further refinement was required. Finally, participants voted electronically to confirm domain placement in the COS (inner/middle/outer/out) and method of assessment. Proceedings were captured in the form of detailed written records and the outcomes of voting.

Results

For peer review only - http://bmjopen.bmj.com/site/about/guidelines.xhtml

Stage 1 Core Domain Set

Stage 1.1: Developing a comprehensive domain list

A total of 57 (episodic) and 58 (chronic) domains were included in the questionnaire, grouped across four areas: symptoms (17), life impact (27 episodic / 28 chronic), treatment effectiveness/ financial impact (10) and complications (2). Piloting informed minor language modifications. Fifty-seven of the domains of interest were included for both episodic and chronic migraine.

Stage 1.2: International modified-Delphi process

<u>*Round one*</u>

Sub-panel 1 (patients) Two organisations advertised the study (Migraine Association, Ireland; National Migraine Centre, UK). Almost 80% (76/96) of patients who expressed an interest in taking part in the study completed the first questionnaire (42/53 CM (79%); 34/43 EM (79%)). Most were female (CM 40/53 (73%); EM 29/43 (66%)) and aged between 36-45 (CM 41%) and 56-65 years (EM 32%) (range 18 to >66 years). Most were from the UK (57%), followed by the US (19%), Ireland (14%), Canada (2%), and the rest of Europe (Denmark (2%), France (5%)).

Sub-panel 2 (professionals) From a total of 198 international healthcare professionals/researchers invited to participate, 64 agreed. Nearly half (31/64 (48%)) joined the panel to complete the episodic migraine questionnaire; slightly more (33/64 (52%)) completed the chronic migraine questionnaires. Most were from the UK 14/33 (42%), with participants from the US 5/33 (15%), Europe (Belgium 1/33 (3%), Germany 2/33 (6%), Italy 1/33 (3%), Netherlands 1/33 (3%), Portugal 1/33 (3%), Serbia 1/33 (3%), Spain 2/33 (6%)) and Turkey 1/33 (3%)), the Russian Federation 1/33 (3%), South Africa 1/33 (3%) and Thailand 1/33 (3%). Professionals included neurologists, nurse specialists, general practitioner, allied health professionals, researchers, and measurement experts (Appendix Table 2).

In total, 75 (64%) and 65 (61%) panellists completed round 1 chronic and episodic migraine questionnaires, respectively.

Most domains were rated as 'important', with few between panel discrepancies. Implementation of the *a priori* decision rule (Appendix Table 1) supported a 50% reduction in domains, with the prioritisation of 18/57 (episodic) and 24/58 (chronic) domains (Table 1).

Table 1. Delphi Round 1 shortlisted domains by voting prioritisation and agreement between panels

Domain	EPISODIC MIGRAINE	CHRONIC MIGRAINE
	Evidence supporting inclusion in Round 2 Delphi	

For peer review only

Section 1: Life impact – symptoms associated with headache / migraine	Voting prioritisation	Qualitative feedback	Voting prioritisation	Qualitative feedback
Cognitive function – difficulty concentrating, ability to 'think clearly' or to remember things	(A)	Yes	A*	
Increased sensitivities – to light, sound, smell, touch	A*		A*	
Pain associated with headache – experience an unpleasant physical sensation that aches or hurts	A**		A*	
Duration of pain associated with a headache	A**		A*	
Frequency of pain associated with a headache	A**		A*	
Severity / intensity of pain associated with a headache	A**		A*	
Physical fatigue – experiencing physical fatigue, tiredness, lacking in energy, feeling physically exhausted	(A)	Yes	A*	
Sleep quality – being able to have a restful sleep	(A)		A*	
Vomiting and/or feelings of nausea	A*		(A)	
Anxiety – concerned, worried, fearful or anxious	(A)	Yes	(A)	Yes
Depressive mood – feeling sad, feeling down, feeling sorry for oneself or feeling depressed	(A)	Yes	(A)	No
Section 2: Life impact – functioning, activities and general wellbeing				
Activities of daily life				
Being able to carry out ***usual tasks or daily activities*** inside or outside the home (not related to paid employment) ***that support an independent lifestyle*** – such as tidying one's home, walking short distances, managing finance, driving, using technology	(A)		A*	
Needing to rest or lie down ***because of*** a headache	(A)		A*	
Emotional wellbeing				
Feelings of isolation – feeling isolated, reduced social interactions	(B)	Yes	(A)	Yes
Self-worth – feeling like a burden to others; can include feeling valued or helpless, accepted or rejected; feelings of self-esteem	(B)	Yes	(A)	Yes
Stress – feelings of distress, frustration or irritation	A*		(A)	Yes
Work/Education				
Being able to carry out ***activities related to work*** (paid or unpaid) / ***study*** to an acceptable or usual standard	A*		A**	
Needing to take ***time-off work*** (paid or unpaid) / ***study***	A*		A*	
Social life				
Social life – relationships with colleagues or peers			A*	
Family roles – being able ***to provide*** usual care and support for family and close friends	(A)	Yes	(A)	Yes
Participation in social or leisure activities – ability to participate in social or leisure activities	(A)	Yes	(A)	Yes
Overall health – an individual's general health status; the ability to live a 'normal ' life	A*		A*	
Self-management – ability to effectively decrease/ minimise / control the impact of migraine on oneself (e.g. pharmacology, diet, lifestyle choices)	A*		A*	

For peer review only

Unpredictability of a migraine – uncertainty of being symptom-free or able to engage in activities	A*	No	(A)	Yes
Trigger factors – ability to avoid / manage migraine trigger factors	(B)	Yes		No
Section 3: Treatment effectiveness and financial impact				
Satisfaction with treatment	A*		A*	
Confidence in treatment	A*		A*	
Consistency of treatment effect	A*		A*	
Medication use – the type (potency) and dose (how much) medication ***taken when experiencing*** a migraine or headache	A*		A*	
Medication use – the type (potency) and dose (how much) medication ***taken to prevent*** a migraine or headache	A*		A*	
Financial impact – the economic cost associated with migraine treatment (to the individual (out of pocket expenses)) and healthcare systems)	(A)		A*	
Use of healthcare resources in response to migraine	(A)		A*	
Section 4: Complications (Adverse Events)				
Treatment side effects – experiencing undesirable secondary effects from taking medications for migraine	A*		A**	
Mortality (death)	(A)		A**	
Included in Round 2 due to importance scores (A** or A*)	18		24	
Included in Round 2 due to qualitative feedback	9		7	
New outcomes added due to qualitative feedback	0		0	
**TOTAL number of outcomes for inclusion in Round 2**	**27**		**31**	

Footnote: Each outcome was assigned to one of six categories reflecting levels of agreement: outcomes classified A** and A* would be included in round 2.

- A** if in both sub-panels the median rating is 9
- A* if in both sub-panels ≥70% rate an outcome ≥7
- (A) if in both sub-panels the median outcome rating is ≥7
- (B) if the median rating for an outcome is ≥7 in only one sub-panel

Qualitative feedback informed further consideration of 10 domains (9 episodic, 7 chronic) not achieving the proposed benchmark. No 'new' domains were proposed.

Round two

Round two questionnaires contained 27 (episodic) and 31 (chronic) domains (Table 1). Round two was completed by 23/33 (70%) and 29/31 (93%) health professionals and 33/42 (79%) and 25/34

For peer review only

For peer review only - http://bmjopen.bmj.com/site/about/guidelines.xhtml

(74%) patients for chronic and episodic migraine, respectively (totalling 54 episodic (83%) and 56 chronic (75%) migraine questionnaires completed).

When prioritised according to the top 10 and top 50% of domains, several overriding ‘meaningful’ domains could be described (Tables 2 a-b); six of which were common to both episodic and chronic migraine: pain, usual activities, cognition, adverse events, overall health, associated symptoms. Respondents to the episodic migraine questionnaire also prioritised self-management, whilst medication use was prioritised by chronic migraine respondents.

Table 2a. Delphi Round 2. Results of domain prioritisation for Episodic Migraine (combined panels n=27)**

Rank*	**Proposed ‘merged’ domain and definition**	**Top 10/27 prioritised domains**	**Top 50% of prioritised domains (rank 1 to 13/27 inclusive)**	**Lower 50% of prioritised domains (rank 14 to 27 inclusive)**
**1**	**Pain**<br>- ***Experience of an unpleasant sensation that aches or hurts in the head; the frequency, severity and duration of this pain is important***	Pain associated with Migraine – experience of an unpleasant sensation that aches or hurts (1/27)		
		Frequency of pain associated with a migraine (2/27)		
		Severity or intensity of pain associated with a migraine (3/27)		
		Duration of pain associated with a migraine (4/27)		
**2**	**Usual activities**<br>- ***Being able to carry out usual activities (including paid or unpaid work, study, domestic chores, care or support for family or close friends) to an acceptable or usual standard***<br>- ***Being able to participate in, or commit to, usual activities***	Being able to carry out ***activities related to work*** (paid or unpaid) ***or study*** to an acceptable or usual standard (5/27)		
			Family roles – able to provide usual care or support for family or close friends, including ability to commit activities (11/27)	
			Needing to take time-off work (paid or unpaid) or study (13/27)	
				Participation in social or leisure activities – ability to participate in, or commit to, social or leisure activities (22/27)
**3**	**Cognition**<br>- ***Difficulty concentrating, ability to ‘think clearly’, or to remember things***	Cognitive function – difficulty concentrating, ability to think ‘clearly’ or to remember things (6/27)		
**4**	**Adverse events**	Treatment side-effects – experiencing undesired secondary effects from taking medications for migraine (7/27)		

For peer review only

**5**	**Overall health**	An individual's general health status; the ability to 'live a normal life' (8/27)		
**6**	**Self-management**	Trigger factors – the ability to avoid / manage migraine trigger factors (9/27)		
			Self-management – ability to effectively decrease / minimise/ control the impact of migraine on oneself (e.g. pharmaceutical, diet, lifestyle choices etc) (11/27)	
				Unpredictability of a migraine – uncertainty of being symptom free or able to engage in activities (17/27) ***** prioritised in top 10 (10/27) by patients***
**7**	**Associated symptoms**	Increased sensitivities – to light, sound, smell or touch (10/27)		
				Vomiting and/ or feelings of nausea (15/27) **** prioritised in top 10 (8/27) by HCPs**
				Physical fatigue – experiencing physical fatigue, tiredness, lacking in energy, feeling physically exhausted (18/27) ***** prioritised in top 50% (11/27) by patients***
8	Medication use			Satisfaction with treatment (14/27) ***** prioritised in top 10 (9/27) by HCPs***
				The type (potency) and dose (how much) of a medication ***taken when experiencing*** a migraine (16/27) ***** prioritised in top 50% (11/27) by HCPs***
				The type (potency) and dose (how much) of a medication ***taken to prevent*** a migraine (21/27)
				Consistency in treatment (23/27)
				Confidence in treatment (25/27)
9	Emotional well-being			Anxiety (19/27)
				Depression (19/27) ***** prioritised in top 50% (13/27) by patients***

For peer review only - http://bmjopen.bmj.com/site/about/guidelines.xhtml

				Stress (24/27)
				Self-worth (24/27)
				Isolation (27/27)

Footnote:
*Top 7 grouped domains – informed by top 10 and top 50% of prioritised domains (13/27).
** 6 domains prioritised differently between the two panels; considered further in Round 3.

Table 2b. Delphi Round 2. Results of domain prioritisation for Chronic Migraine (combined panels n=31)**

Rank*	**Domain and definition**	**Top 10/31 prioritised domains**	**Top 50% of prioritised domains (rank 1 to 15/31 inclusive)**	**Lower 50% of prioritised domains (rank 16 to 31 inclusive)**
**1**	**Pain**<br>- *Experience of an unpleasant sensation that aches or hurts in the head; the frequency, severity and duration of this pain is important*	Severity or intensity of pain associated with a migraine (1/31)		
		Pain associated with Migraine – experience of an unpleasant sensation that aches or hurts (2/31)		
		Frequency of pain associated with a migraine (3/31)		
		Duration of pain associated with a migraine (4/31)		
**2**	**Usual activities**<br>- *Being able to carry out usual activities (including paid or unpaid work, study, domestic chores, care or support for family or close friends) to an acceptable or usual standard*	Being able to carry out usual tasks or daily activities inside or outside the home (not related to paid employment) *that support an independent lifestyle* – such as tidying one's home, walking short distances, managing finance, driving, usual technology *(instrumental activities of daily life)* (5/31)		
	- *Being able to participate in, or commit to, usual activities*	Being able to carry out activities related to work (paid or unpaid) or study to an acceptable or usual standard (6/31)		
			Needing to take time-off work (paid or unpaid) or study (11/31)	
				Family roles – able to provide usual care or support for family or close friends, including ability to commit activities (19/31)
				Participation in social or leisure activities – ability to participate in, or commit to, social or leisure activities (22/31)
**3**	**Cognition**<br>- *Difficulty concentrating, ability to 'think clearly', or to remember things*	Cognitive function – difficulty concentrating, ability to think 'clearly' or to remember things (7/27)		
**4**	**Adverse events**	Treatment side-effects – experiencing undesired		

For peer review only - http://bmjopen.bmj.com/site/about/guidelines.xhtml

		secondary effects from taking medications for migraine (8/31)		
				Mortality (death) (26/31) ***** prioritised in top 50% (15/31) by HCPs***
**5**	**Associated symptoms**	Increased sensitivities – to light, sound, smell or touch (9/31)		
		Physical fatigue – experiencing physical fatigue, tiredness, lacking in energy, feeling physically exhausted (10/31)		
			Sleep quality – being able to have a restful sleep (14/31)	
			Needing to rest or lie down because of a headache (15/31)	
**6**	**Medication use**		Satisfaction with treatment (12/31)	
				The type (potency) and dose (how much) of a medication taken to prevent a migraine (21/31)
				Consistency in treatment effect (23/31)
				The type (potency) and dose (how much) of a medication taken during a migraine (24/31)
				Confidence in treatment (28/31)
**7**	**Overall health**		An individual's general health status; the ability to 'live a normal life' (13/31)	
8	Emotional well-being			Stress – feelings of distress, frustration or irritation (16/31) ***** prioritised in top 10 (10/31) by HCPs***
				Anxiety – concerned, worried, fearful or anxious (20/31)
				Self-worth – feeling like a burden to others; can include feeling valued or helpless; accepted or rejected; feelings of self-esteem (28/31)
				Feelings of isolation – feeling isolated; reduced social interactions (29/31)
				Social role – relationship with work colleagues or peers (31/31)
9	Self-management			Self-management – ability to effectively decrease / minimise/ control the impact of migraine on oneself (e.g.

For peer review only - http://bmjopen.bmj.com/site/about/guidelines.xhtml

				pharmaceutical, diet, lifestyle choices etc) (17/31)
				Unpredictability of a migraine – uncertainty of being symptom free or able to engage in activities (18/31) ***** prioritised in top 50% (14/31) by patients***
10	Financial impact			Economic cost associated with treatment for headache (to the individual (out-of-pocket expenses) and healthcare system) (25/31)
				Use of healthcare resources in response to headache (30/31)

Footnote:
* Top 5 grouped domains – informed by top 10 prioritised domains. Top 7 grouped domains – informed by top 13 and top 50% of prioritised domains (15/31).
** 3 domains prioritised differently between the two panels; considered further in Round 3.

Sub-panel discrepancies for both episodic and chronic migraine included patients' prioritisation of overall health, physical fatigue, unpredictability, and self-management. Patients with episodic migraine also prioritised emotional wellbeing. Although awarded fewer points, people with chronic migraine prioritised the importance of social role and emotional wellbeing. In contrast, healthcare professionals prioritised treatment satisfaction, treatment side-effects and vomiting/ nausea for episodic migraine, and mortality and stress for chronic migraine.

Round three

Round three was completed by 23/23 (100%) and 21/29 (72%) health professionals, and 29/33 (88%) and 23/25 (92%) patients for chronic and episodic migraine, respectively (totalling 52/56 (93%) for chronic migraine and 44/54 EM (81%) for episodic migraine. Six and three domain discrepancies (top 10 or top 50% for one sub-panel only) were considered for episodic migraine (treatment satisfaction; vomiting/ feelings of nausea; medication taken during a migraine; unpredictability; physical fatigue; depressive mood) and chronic migraine (stress; mortality; unpredictability), respectively (Appendix Table 3).

The seven domains for episodic migraine were retained (>76% across sub-panels; >84% combined) (Table 3) and a new domain 'Treatment Satisfaction' proposed (>70% healthcare professionals; 68% combined) (Appendix Table 3). Voting on sub-panel discrepancies further supported the inclusion of vomiting/feelings of nausea, physical fatigue, and depressive mood within the developing core

For peer review only - http://bmjopen.bmj.com/site/about/guidelines.xhtml

domain set for episodic migraine (Appendix Table 3). Qualitative feedback in the questionnaire supported a more positive re-phrasing of the concept of self-management.

Table 3. Delphi Round 3: results of voting for domains for episodic and chronic migraine

Proposed CORE DOMAINS for EM and CM<br>*(For voting in Round 3)*			EPISODIC MIGRAINE<br>*Voting*			CHRONIC MIGRAINE<br>*Voting*		
Prioritised domains<br>*(informed by Round 2)*	Proposed 'Meaningful Domain' and definition<br>*(bold text informed by R3 qualitative feedback)*	Q	Patient<br>(n=23)	HCPs<br>(n=21)	Combined<br>(n=44)	Patient<br>(n=29)	HCPs<br>(n=23)	Combined<br>(n=52)
• Pain associated with migraine – an unpleasant sensation that aches or hurts<br>• Frequency of pain associated with migraine<br>• Severity or intensity of pain associated with migraine<br>• Duration of pain associated with migraine	***PAIN***<br>- Experience of an unpleasant sensation in the head that aches or hurts and is associated with experiencing a migraine;<br>- the components of frequency, severity and duration of pain are all important<br><br>*Qualitative feedback supported the addition of:*<br>- *unpleasant sensation in the head* ***… face, neck and/or shoulders …***	a.<br><br>b.	100.0%<br><br>82.6%	100.0%<br><br>100.0%	100.0%<br><br>90.9%	96.6%<br><br>89.7%	86.9%<br><br>95.7%	92.3%<br><br>92.3%
• An individual's health status; the ability to live a 'normal' life	**OVERALL HEALTH**<br>- An individual's health status; the ability to live a 'normal' life<br><br>*Qualitative feedback challenged the concept or 'normal life' and the lack of clarity re a focus on* ***migraine-specific or general quality of life.*** *To be explored during the consensus meeting.*	a.<br><br>b.	100.0%<br><br>87.0%	90.5%<br><br>81.0%	95.5%<br><br>84.1%	96.6%<br><br>89.7%	87.0%<br><br>78.3%	92.3%<br><br>84.6%
• Being able to carry out *activities related to work (paid or unpaid) or study* to an acceptable or usual standard<br>• Family roles- able to provide usual care or support for family or close friends,	**USUAL ACTIVITIES**<br>- Being able to carry out usual activities (including paid or unpaid work, study, domestic chores, family or leisure activities, care or support for family or close friends) to an acceptable or usual standard<br>- Being able to participate in or	a.<br><br>b.	95.7%<br><br>95.7%	81.0%<br><br>76.2%	88.6%<br><br>86.4%	100.0%<br><br>89.7%	95.7%<br><br>95.7%	98.1%<br><br>92.3%

For peer review only

For peer review only - http://bmjopen.bmj.com/site/about/guidelines.xhtml

including to commit to activities *(EM only)*<br>• Need to take time-off work (paid or unpaid) or study<br>• Being able to carry out usual tasks or daily activities inside or outside the home (not related to employment) *that support an independent lifestyle* – such as tidying one's home, walking short distances, managing finance, driving, using technology *(CM only)*	commit to usual activities<br><br>*Qualitative feedback supported the importance of including 'unpredictability' in the definition:*<br>- ***Being able to plan, commit to, or participate in usual activities, including work, usual social or caring roles (due to the unpredictability of a migraine)***							
• Cognitive function – difficulty concentrating, ability to think 'clearly' or to remember things	**COGNITIVE FUNCTION**<br>- Difficulty with concentrating, thinking clearly, or remembering things;<br><br>*Qualitative feedback supported the addition of:*<br>- ***difficulty with communication (word finding, slow or slurred speech)***	a.<br><br>b.	95.7%<br><br>91.3%	100.0%<br><br>90.5%	97.7%<br><br>90.9%	96.6%<br><br>93.1%	95.7%<br><br>95.7%	96.1%<br><br>94.2%
• Treatment side-effects – experiencing undesired secondary effects from taking medications for migraine	**ADVERSE EFFECTS**<br>- Experiencing undesired secondary effects from taking medications for migraine<br><br>*Qualitative feedback supported adoption of the CTCAE standardised definition of adverse events:*<br>- ***'any unfavourable and unintended sign, symptom, or disease temporarily associated with the use of a medical treatment or procedure that may or may not be considered related to the medical treatment or***	a.<br><br>b.	100.0%<br><br>87.0%	100.0%<br><br>90.5%	100.0%<br><br>88.6%	89.7%<br><br>93.1%	95.7%<br><br>82.6%	92.3%<br><br>88.5%

For peer review only - http://bmjopen.bmj.com/site/about/guidelines.xhtml

	procedure.' (CTCAE ref)							
• Increased sensitivities – to light, sound, smell or touch<br>• Physical fatigue – experiencing physical fatigue, tiredness, lacking in energy, feeling physically exhausted *(CM ony)*<br>• Sleep quality – being able to have a restful sleep *(CM only)*<br>• Needing to rest or lie down because of a headache *(CM only)*	**ASSOCIATED SYMPTOMS**<br>- Increased sensitivities – to light **(photophobia)**, sound **(phonophobia)**, smell, touch, or movement<br>- Physical fatigue – experiencing physical fatigue, tiredness, lacking in energy, feeling physically exhausted (CM only)<br>- Sleep quality – being able to have a restful sleep (CM only)<br>- Needing to rest or lie down because of a headache (CM only)<br><br>*Qualitative feedback highlighted concern over the omission of the following components from associated symptoms:*<br>- *Visual disturbances.*<br>- *Depressive mood*<br>- *Vomiting / feelings of nausea*<br>*All to be explored in consensus meeting (for both EM and CM)*	a.<br>b.	87.0%<br>87.0%	100.00%<br>90.5%	93.2%<br>88.6%	96.6%<br>93.1%	73.9%<br>73.9%	86.5%<br>84.6%
• Satisfaction with treatment	**MEDICATION USE**<br><br>**Voting: Proposed domain REJECTED (values < 70%)**<br><br>Qualitative feedback highlighted the importance of a domain that was not just focused on medication use.<br><br>*NOTE: Voting on sub-group discrepancies (Table R3b) supported the inclusion of 'Treatment Satisfaction' as a domain within the EM domain set.*<br>*Core group recommendation that 'TREATMENT SATISFACTION' is explored in consensus meeting for both EM and CM*	a.<br>b.	N/A	N/A	N/A	79.3%<br>72.4%	**69.6%**<br>**60.9%**	75.0%<br>**67.3%**
	**SELF-MANAGEMENT**							

For peer review only - http://bmjopen.bmj.com/site/about/guidelines.xhtml

• Trigger factors – the ability to avoid / manage migraine trigger factors<br>• Self-management – the ability to effectively decrease / minimise / control the impact of migraine on oneself (e.g. by pharmaceutical, diet, lifestyle choices etc.)	- Ability to effectively decrease / minimise / control the impact of migraine on oneself (e.g. by pharmaceutical, diet, lifestyle choices etc.)<br>- Ability to avoid / manage migraine trigger factors<br><br>*Qualitative feedback – proposed a more positive definition:*<br>- ***Living better with migraine through lifestyle, dietary, pharmaceutical choices and taking an active part in long-term management of migraine with education and support.***<br>- ***Enabling patients to become active partners in their migraine treatment***	a.<br><br>b.	95.7%<br><br>91.3%	85.7%<br><br>81.0%	90.9%<br><br>86.4%	N/A	N/A	N/A

Footnote:

Participants were invited to vote (Yes/No): a. Are you happy with the grouping of prioritised domains (Yes/No)? ; b. Are you happy with the proposed 'meaningful' domain and definition (Yes/No)?

N/A: Not applicable. Panellists did not vote in this domain.

For peer review only

Six of the seven domains for chronic migraine were retained (>73% across sub-panels; >80% combined) (Table 3). 'Medication Use' was rejected (<70%), and a redefining as 'Treatment Satisfaction' proposed. Qualitative feedback also highlighted the omission of 'visual disturbances' from 'Associated Symptoms', and the movement of 'Sleep Quality' to 'Usual Activities'.

For both episodic and chronic migraine, qualitative feedback highlighted the importance of communication difficulties within cognitive function; further consideration of vomiting/nausea, fatigue and depressive mood as additional 'Associated Symptoms'; and unpredictability and ability to uphold usual commitments within 'Usual Activities'. Further clarification of the concept of 'Overall

For peer review only - http://bmjopen.bmj.com/site/about/guidelines.xhtml

Health' – for example, general or migraine-specific health, was proposed and adoption of a standardised definition of 'adverse events' (Common Terminology Criteria for Adverse Events (CTCAE) [28].

The process defined seven core domains common to episodic and chronic migraine (Table 3). Additionally, episodic migraine included 'self-management'.

Stage 2: Core Measurement Set

International expert panel face-to-face meeting

The one-day meeting took place at Warwick University in December 2018. Seven patients (three with episodic migraine and four with chronic migraine) and seven healthcare professionals/researchers (two doctors, two nurses, one physiotherapist, two measurement experts) participated from two countries (UK, Portugal). Ten core group members, including two patient research partners (GP, BB), attended.

Pain – was re-defined as migraine-specific pain and endorsed as an inner core domain for episodic and chronic migraine (>70%) (Table 4; Figure 2). Based on review of existing measures and group discussion voting supported recommendation of the 11-point numerical rating scale (NRS) for assessing pain intensity [29] and number of headache/migraine days per month for pain frequency [1, 22]. Due to the complexities around the concepts of headache and migraine, it was recommended that the specific terminologies should be defined by individual studies.

Table 4. Consensus meeting: results from small and large group discussions and voting.

Domain	Small group	Large group	Final decision [a]
**Pain**	***Domain***<br>Voting supported inclusion of Pain for EM and CM (>70%)<br>Three aspects of Pain included:<br>• intensity (11/11)<br>• frequency (10/11)<br>• duration (8/11)<br>Proposed domain refinement to 'Migraine-specific Pain'<br><br>***Measurement***<br>Voting for individual options did not exceed 70%<br>Preferred assessments:<br>Intensity: 11-point NRS (55%)<br>Frequency: Number of headache/migraine days (64%)<br>Duration: Cumulative hours per 28-days of moderate/severe pain (55%)	***Domain***<br>INNER core: Migraine-specific pain (no further voting required)<br><br>***Measurement***<br>Pain intensity: 11-point NRS (80%)<br><br>Pain frequency: Number of headache/migraine days (>70%)<br><br>Pain duration: No consensus. Proposed that daily capture (using paper or electronic diary) or retrospective capture using a questionnaire may not be feasible for all trials.<br>Voting: MIDDLE circle (89%)	***Domain – both EM and CM***<br>INNER core : Migraine-specific pain<br>Components: intensity and frequency<br><br>***Measurement***<br>Pain intensity – 11-point NRS (anchors 'no pain' and 'pain as bad as you can imagine')<br><br>Pain frequency<br>• number of headache / migraine days<br><br>Pain Duration: MIDDLE circle: important but not feasible for all trials / research studies

For peer review only - http://bmjopen.bmj.com/site/about/guidelines.xhtml

**Overall Health**	***Domain***<br>Voting supported redefining domain as Migraine-specific Quality of Life (73%)<br><br>***Measurement***<br>Migraine Functional Impact Questionnaire (72%)	***Domain***<br>INNER core: Migraine-specific Quality of Life (no further voting required)<br><br>***Measurement***<br>Migraine Functional Impact Questionnaire	***Domain – both EM and CM***<br>INNER core: Migraine-specific Quality of Life<br><br>***Measurement***<br>Migraine Functional Impact Questionnaire
**Adverse Events**	***Domain***<br>Voting supported the rejection of adverse events from the core domain set (82%)<br><br>***Measurement***<br>N/A	***Domain***<br>Recommendations were supported. Should be captured as part of good clinical practice guidance.	Not included in the COS for EM or CM
**Self-management**	***Domain***<br>No consensus on the inclusion (46%) / exclusion (54%) of self-management. Participants considered it to be important to both EM and CM, but requiring greater conceptualisation before it can be accurately measured	***Domain***<br>Group confirmed the importance of self-management for both EM and CM, but agreed that the lack of conceptualisation and method of assessment prevented inclusion in the COS.<br>Voting: RESEARCH AGENDA (73%)	***Domain and measurement – both EM and CM***<br>OUTER circle - Research Agenda: important but requiring further study
**Cognitive function**	***Domain***<br>Voting supported the rejection of cognitive function as a separate core domain (70%)<br><br>But participants supported cognitive function as an important concept.	***Domain***<br>Recommendations supported. The importance of cognitive function was supported and the potential for it to be captured with migraine-specific quality of life proposed.	Not included as a separate core domain for EM or CM.<br><br>Cognitive function is included within the new domain 'Migraine-specific Quality of Life' and will be assessed by the MFIQ
**Associated symptoms**	***Domain***<br>No consensus on the inclusion (50%) / exclusion (50%) of associated symptoms.<br><br>Participants discussed the importance of a wide range of associated symptoms – but capture of all would not be feasible in all trials (and hence not core)	***Domain***<br>Participants recognised pain as an important 'associated symptom' and the inclusion of several additional associated symptoms within the new domain 'MQoL' (captured by the MFIQ).<br><br>Capturing a larger number of associated symptoms, or specific additional symptoms - such as fatigue - should be study specific and not core.<br>Voting: MIDDLE circle (100%)	***Domain and measurement – both EM and CM***<br>MIDDLE circle: important but not feasible to include in all trials / research studies.
**Usual activities**	***Domain***<br>Voting supported the inclusion as a component of a new domain 'MQoL' (100%)<br><br>***Measurement***<br>Usual activities, as a component of MQoL to be assessed with the MFIQ (80%)	***Domain***<br>Recommendations were supported<br><br>***Measurement***<br>N/A	Not included as a separate core domain for EM or CM.<br><br>Usual activities is included within the new domain 'Migraine-specific Quality of Life' and will be assessed by the MFIQ
**Treatment satisfaction**	***Domain***	***Domain***	***Domain and measurement – both EM and CM***

For peer review only

For peer review only - http://bmjopen.bmj.com/site/about/guidelines.xhtml

	Considered important – but no consensus on the inclusion (64%) / exclusion(36%) of treatment satisfaction due to need for greater clarity	Group confirmed the importance of treatment satisfaction for both EM and CM, but agreed that the lack of conceptualisation and method of assessment prevented inclusion in the COS<br>Voting: RESEARCH AGENDA (100%)	OUTER circle - Research Agenda: important but requiring further study

Footnote: [a] *Core 'inner' circle:* domain is unambiguous with an acceptable method of assessment; *Middle circle:* domain is important, but not feasible for all preventative trials and research studies; *Outer circle*: domain is important, but requires further study (research agenda) – e.g. lacks conceptual clarity or method of assessment.

Overall health – was re-defined as 'migraine-specific quality of life' (MSQoL), endorsed as an inner core domain for both episodic and chronic migraine (Table 4; Figure 2). Presented with evidence for generic and migraine quality of life measures, participants preferred the Migraine Functional Impact Questionnaire (MFIQ) [2, 30]. The four domain scores of the MFIQ address several key concepts highlighted throughout the COSMIG process – including usual activities, physical, cognitive, social, and emotional function. It also provides a global item score for usual activities.

Pain duration and ***associated symptoms*** were both judged as important, but not feasible for inclusion in all trials/research studies and placed in the middle circle (Table 4; Figure 2).

Self-management and ***Treatment satisfaction*** – were both considered important for both episodic and chronic migraine, but lack of conceptualisation and assessment supported their placement on the research agenda (outer circle) (Table 4; Figure 2).

Cognitive function and ***Usual activities*** were both rejected as independent core domains but proposed as important components of migraine-specific quality of life (Table 4).

Adverse events – was rejected as a core domain, with the proposition that such reporting should be part of good clinical practice guidance (Table 4; Figure 2).

The result was a two domain Core Outcome Set for both EM and CM (COSMIG) (Table 4; Figure 2):

1) Migraine-specific pain: intensity assessed with the 11-point NRS and frequency as the number of headache/migraine days over a specified period; and

2) Migraine-specific quality of life – assessed with the MFIQ [30].

Discussion

For peer review only - http://bmjopen.bmj.com/site/about/guidelines.xhtml

The COSMIG process has identified two core domains - pain and migraine-specific quality of life – that are recommended as part of *a priori*-designated outcomes in future preventive intervention clinical trials for both episodic and chronic migraine. Pain assessment should include both intensity measured with an 11-point NRS, and frequency assessed as the number of headache/migraine days per 28 days. Migraine-specific quality of life should be assessed with the Migraine Functional Impact Questionnaire (MFIQ) [30]. Complex concepts around headache and migraine meant that participants in the consensus meeting were not able to make recommendations for the phrasing of questions on pain severity (e.g., worst, average or typical) or the definition of a migraine/headache day. Thus, the specific terminologies should be defined, and reported, by the needs of individual studies. Likewise, the specific timing of assessments should be driven by the requirements of the study.

Participants in the consensus meeting preferred the MFIQ over other measures of migraine-related quality of life such as the Migraine Specific Quality-of-Life Questionnaire MSQv2.1 and the 6-item Headache Impact Test (HIT-6) because participants, in particular patient participants, felt its domains best reflected the impact migraine has on people's lives. This matches the aims of the original developers who specifically sought to address gaps in existing patient reported outcomes [31]. A licence is needed to use the MFIQ available from Legal@evidera.com. The owners advise us that it will be available free of charge for non-commercial research (email Evidera 15 May 2020, personal communication). Pain duration and associated symptoms are important but are not considered core. How to assess self-management and treatment satisfaction requires further research before recommendations can be made.

Our recommendation to include a reduction in the severity (intensity) and frequency in migraine pain is further supported by a recent modified-Delphi study conducted in the US, which sought to identify outcomes for value-based contracting for migraine medications.[32] However, a Delphi study of experts (N=12) published after our work was completed focussed on establishing the most useful outcome measures, specifically for non-pharmacological interventions for migraine, identified the Migraine Disability Assessment (MIDAS) followed by the HIT-6 as preferred outcomes.[33] Our empirical work does not support this prioritisation of outcome measures, [2,34]

The COSMIG recommendations contrast with previous guidance for trials of prophylaxis in chronic migraine that recommend a single primary outcome derived from headache/migraine days. Patient-reported headache-related quality of life appears last in order of the secondary outcomes[1] and guidelines for trials of prophylaxis in episodic migraine do not include quality of life as an outcome [13]. Informed by current good practice guidance in core outcome set development [9, 14], this study included international participation from patient and professional panellists in an on-line Delphi study and a

For peer review only - http://bmjopen.bmj.com/site/about/guidelines.xhtml

subsequent face-to-face meeting. All data pertaining to the Delphi study were analysed both separately and combined to ensure that the views of sub-panels were clearly reported. This approach highlighted the value placed upon patient-reported outcomes such as pain and quality of life by patients and health professionals. However, discrepancies pertaining to, for example, the importance of fatigue, unpredictability, emotional impact, and cognitive function were described. Such discrepancies have been reported in other long-term musculoskeletal conditions[35] and more recently in a survey of health professionals and patients with COVID.[36] Evidence of such discrepancies is a key driver for the suggestion that patients' views are given at least equal wight to those of professionals in the process of core outcome set development.[9] Incorporating outcomes that have resonance to all stakeholders can enhance trial relevance, providing valued information to inform decision-making in clinical practice and health policy settings.

Whilst individuals from 14 countries were included in the Delphi study, participants from just two countries (England and Portugal) contributed to the face-to-face meeting. However, both the Delphi process and consensus meeting sought input from credible 'experts'.[17, 19] For patients, expert is defined by experience of living with chronic or episodic migraine, and for health professionals by their relative expertise in migraine-related research. The wide international involvement throughout the Delphi study improved international reach and helped ensure a wider relevance of the recommendations. We note that Delphi results are obtained from inviting experts to join a panel; as this eschews sampling, no inference should be made to any larger definable population.

Active pre-engagement with potential participants in the Delphi study enabled targeted follow-up of non-responders in round one [37]. We note that the participation rate of invited panellists is higher than reported in some other Delphi studies, where response rates between 30 and 40% have been reported.[21] Moreover, a recent international Delphi study which sought to reach agreement on outcome measures for assessing the effectiveness of non-pharmacological interventions in migraine invited just 35 eligible researchers as subject experts, and four patients.[33] Of the researchers, just 12 agreed to participate, with 10 (28%) completing all three rounds. This suggests that the focus of our Delphi study resonated with panellists, and moreover, retention across subsequent rounds was high, with responses from both sub-panels exceeding 70%.

More people with chronic migraine than with episodic migraine participated in the Delphi study, sub-panel responses were analysed separately for both panels. Seven of the eight prioritised domains were common to both episodic and chronic migraine; self-management was unique to

For peer review only - http://bmjopen.bmj.com/site/about/guidelines.xhtml

episodic migraine. However, participants in the consensus meeting agreed that whilst poor conceptualisation and lack of assessment option prevented its consideration as a core domain, self-management was important for both episodic and chronic migraine.

We relied on participant self-identification of diagnosis of episodic/chronic migraine. Any misclassification is unlikely to have any substantive impact on our findings. The study included a broad age-range of patient participants. Similarly, the healthcare professionals involved had a broad spectrum of experience in the care of patients with migraine and in migraine-related research.

Working collaboratively with patient research partners throughout the research contributed to the crafting of 'meaningful' domains at each stage of the Delphi process, giving validity to the proposed lists [20]. The initial Delphi questionnaire provided a comprehensive reflection of domains that might be assessed in chronic or episodic migraine. Additional domains were not proposed by participants in round one, supporting the comprehensiveness and relevance of content. Patient partners checked the comprehensibility and relevance of short-listed methods of assessment presented to participants in the consensus meeting, contributing to the debate and supporting lay participants during group discussions. All patient partners contributed to manuscript edits throughout the write-up phase.

The recommended COSMIG core set should be complemented by additional trial outcomes pertinent to the particular intervention being evaluated[37]. However, standardisation of core data collection is strongly advised to reduce the potential for systematic bias and enhance the quality of patient-reported outcomes data [8, 9]. More work is now needed on how to evaluate the self-management and treatment satisfaction domains.

Through an international collaboration between patients, researchers, and health professionals, we have facilitated consensus on a Core Outcome Set for reporting on preventative intervention trials and research studies in adults with episodic or chronic MIGraine (COSMIG). We recommend that both pain (intensity and frequency) and migraine-specific quality of life are included as core domains. To support meaningful comparisons across studies, we recommend that pain intensity be assessed with a NRS [29] and frequency by determining the number of migraine days; migraine-specific quality of life should be assessed with the MFIQ [30]. The timing of assessments should be determined by individual studies.

For peer review only - http://bmjopen.bmj.com/site/about/guidelines.xhtml

Acknowledgements

We are very grateful to members of the International Steering Group (Richard Lipton (RL), Rigor Jensen (RJ)) and the core COSMIG group (MU, MM, Brendan Davies, RP, SPe, BB, GP, LM, RL, RJ, Viv Nichols (VN), Shilpa Patel (SPa), KS) for their oversight of the study. We are very grateful to everyone who participated in the Delphi study and attended the consensus meeting, and to Spa and VN for their expert facilitation during the consensus meeting.

Competing interests

MU and RF are directors and shareholders of Clinvivo Ltd. MU recused himself from any discussions related to the choice of Delphi platform for this study. MU is chief investigator or co-investigator on multiple previous and current research grants from the UK National Institute for Health Research, Arthritis Research UK and is a co-investigator on grants funded by the Australian NHMRC. He is an NIHR Senior Investigator. He has received travel expenses for speaking at conferences from the professional organisations hosting the conferences. MU and RF are part of an academic partnership with Serco Ltd related to return to work initiatives. MU is a co-investigator on two NIHR funded studies receiving additional support from Stryker Ltd. He has accepted honoraria for teaching/lecturing from CARTA. He was an editor of the NIHR journal series, and a member of the NIHR Journal Editors Group, for which he received a fee.

MSM serves on the advisory board for Abbott, Allergan, Eli Lilly, Medtronic, Novartis, TEVA; has received payment for the development of educational presentations from Allergan, electroCore, Eli Lilly, Medtronic, Novartis, and TEVA; and, has received research grants from Abbott, electroCore and Medtronic

SPa is a director of Health Psychology Services Ltd which, in part, provides psychological treatments for those with chronic pain.

No other competing interests declared.

Funding Statement

For peer review only

For peer review only - http://bmjopen.bmj.com/site/about/guidelines.xhtml

This study was funded by the National Institute for Health Research (NIHR) Programme Grants for Applied Research programme (RP-PG-1212-20018).The views expressed are those of the author(s) and not necessarily those of the NIHR or the Department of Health and Social Care.

Active collaboration with our patient research partners was supported by a 'Delivering Results Award' (IAS/23022/16) from the Institute of Advanced Studies, Warwick University.

Data sharing statement

De-identified data will be shard through the university accessible databases or repositories at Warwick University. Please contact Dr KH is additional information is required: email k.l.haywood@warwick.ac.uk

Authors' contribution

KH, MM, MU, RP, RF, RL, SPe, BB, LM and GP made substantial contributions to the conception and design of the study. KH, MM, RF, RP, RL, SPe, BB, LM, GP and MU made substantial contributions to developing the protocol. KH, MM, MU, RP, RF, RL, RB, SPe, AL, KS, BB, LM and GP made substantial contributions to the acquisition of data, analysis and interpretation of data. All authors have been involved in drafting the manuscript or revising it critically for important intellectual content; given final approval of the version to be published.

Ethics statement

Ethical approval was gained from Warwick Medical School Biomedical and Scientific Research Ethics Committee REGO-2017-1921

For peer review only

For peer review only - http://bmjopen.bmj.com/site/about/guidelines.xhtml

References

1. Tassorelli C, Diener HC, Dodick DW, et al. Guidelines of the International Headache Society for controlled trials of preventive treatment of chronic migraine in adults. *Cephalalgia : an international journal of headache* 2018; 38: 815-832. 2018/03/06. DOI: 10.1177/0333102418758283.
2. Haywood KL, Mars TS, Potter R, et al. Assessing the impact of headaches and the outcomes of treatment: A systematic review of patient-reported outcome measures (PROMs). *Cephalalgia : an international journal of headache* 2018; 38: 1374-1386. 2017/09/19. DOI: 10.1177/0333102417731348.
3. Probyn K, Bowers H, Caldwell F, et al. Prognostic factors for chronic headache: A systematic review. *Neurology* 2017; 89: 291-301. 2017/06/16. DOI: 10.1212/wnl.0000000000004112.
4. Clarke M. Standardising outcomes for clinical trials and systematic reviews. *Trials* 2007; 8: 39. DOI: 10.1186/1745-6215-8-39.
5. Kirkham JJ, Gargon E, Clarke M, et al. Can a core outcome set improve the quality of systematic reviews?--a survey of the Co-ordinating Editors of Cochrane Review Groups. *Trials* 2013; 14: 21. 2013/01/24. DOI: 10.1186/1745-6215-14-21.
6. Jackson JL, Cogbill E, Santana-Davila R, et al. A Comparative Effectiveness Meta-Analysis of Drugs for the Prophylaxis of Migraine Headache. *PLoS One* 2015; 10: e0130733. 2015/07/15. DOI: 10.1371/journal.pone.0130733.
7. Silberstein SD, Lipton RB, Dodick DW, et al. Efficacy and safety of topiramate for the treatment of chronic migraine: a randomized, double-blind, placebo-controlled trial. *Headache* 2007; 47: 170-180. 2007/02/16. DOI: 10.1111/j.1526-4610.2006.00684.x.
8. Kirkham JJ, Dwan KM, Altman DG, et al. The impact of outcome reporting bias in randomised controlled trials on a cohort of systematic reviews. *BMJ (Clinical research ed)* 2010; 340: c365. 2010/02/17. DOI: 10.1136/bmj.c365.
9. Williamson PR, Altman DG, Bagley H, et al. The COMET Handbook: version 1.0. *Trials* 2017; 18: 280. 2017/07/07. DOI: 10.1186/s13063-017-1978-4.
10. Williamson PR, Altman DG, Blazeby JM, et al. Developing core outcome sets for clinical trials: issues to consider. *Trials* 2012; 13: 132. DOI: 10.1186/1745-6215-13-132.
11. Gorst SL, Gargon E, Clarke M, et al. Choosing Important Health Outcomes for Comparative Effectiveness Research: An Updated Review and User Survey. *PLOS ONE* 2016; 11: e0146444. DOI: 10.1371/journal.pone.0146444.
12. Tunis SR, Clarke M, Gorst SL, et al. Improving the relevance and consistency of outcomes in comparative effectiveness research. *Journal of comparative effectiveness research* 2016; 5: 193-205. 2016/03/02. DOI: 10.2217/cer-2015-0007.
13. Tfelt-Hansen P, Pascual J, Ramadan N, et al. Guidelines for controlled trials of drugs in migraine: third edition. A guide for investigators. *Cephalalgia : an international journal of headache* 2012; 32: 6-38. 2012/03/03. DOI: 10.1177/0333102411417901.
14. Boers M, Kirwan JR, Wells G, et al. Developing core outcome measurement sets for clinical trials: OMERACT filter 2.0. *Journal of clinical epidemiology* 2014; 67: 745-753. 2014/03/04. DOI: 10.1016/j.jclinepi.2013.11.013.
15. Ward F, Popay J, Porroche-Escudero A, et al. Mainstreaming public involvement in a complex research collaboration: A theory-informed evaluation. 2020; n/a. DOI: 10.1111/hex.13070.

For peer review only - http://bmjopen.bmj.com/site/about/guidelines.xhtml

16. Nichols VP, Ellard DR, Griffiths FE, et al. The lived experience of chronic headache: a systematic review and synthesis of the qualitative literature. 2017; 7: e019929. DOI: 10.1136/bmjopen-2017-019929 %J BMJ Open.
17. Murphy MK, Black NA, Lamping DL, et al. Consensus development methods, and their use in clinical guideline development. *Health Technol Assess* 1998; 2: i-iv, 1-88. 1998/04/30.
18. Helmer O and Dalkey N. An experimental application of the Delphi Method to the use of experts. *The Rand Corporation Memorandum RM 727/1 – Abridged July 2961,* 1962.
19. Fink A, Kosecoff J, Chassin M, et al. Consensus methods: characteristics and guidelines for use. *Am J Public Health* 1984; 74: 979-983. 1984/09/01. DOI: 10.2105/ajph.74.9.979.
20. Orbai AM, de Wit M, Mease P, et al. International patient and physician consensus on a psoriatic arthritis core outcome set for clinical trials. *Annals of the rheumatic diseases* 2017; 76: 673-680. 2016/09/11. DOI: 10.1136/annrheumdis-2016-210242.
21. Prinsen CAC, Vohra S, Rose MR, et al. How to select outcome measurement instruments for outcomes included in a "Core Outcome Set" – a practical guideline. *Trials* 2016; 17: 449. DOI: 10.1186/s13063-016-1555-2.
22. International Headache Society. ICHD / Guidelines, http://www.ihs-headache.org/ichd-guidelines (April 2020).
23. The National Institute of Neurological Disorders and Stroke (NINDS). Common Data Standards - Headache, https://www.commondataelements.ninds.nih.gov/headache (accessed 01/04/2020).
24. Dworkin RH, Turk DC, Farrar JT, et al. Core outcome measures for chronic pain clinical trials: IMMPACT recommendations. *Pain* 2005; 113: 9-19. 2004/12/29. DOI: 10.1016/j.pain.2004.09.012.
25. Turk DC, Dworkin RH, Allen RR, et al. Core outcome domains for chronic pain clinical trials: IMMPACT recommendations. *Pain* 2003; 106: 337-345. 2003/12/09. DOI: 10.1016/j.pain.2003.08.001.
26. IMMPACT. Initiative on Methods, Measurement, and Pain Assessment in Clinical Trials, http://www.immpact.org/ (accessed April 2020).
27. Tugwell P, Boers M, Brooks P, et al. OMERACT: an international initiative to improve outcome measurement in rheumatology. *Trials* 2007; 8: 38. 2007/11/28. DOI: 10.1186/1745-6215-8-38.
28. Institute NC. Common Terminology Criteria for Adverse Events (CTCAE), https://ctep.cancer.gov/protocoldevelopment/electronic_applications/ctc.htm (accessed April 2020).
29. Hjermstad MJ, Fayers PM, Haugen DF, et al. Studies comparing Numerical Rating Scales, Verbal Rating Scales, and Visual Analogue Scales for assessment of pain intensity in adults: a systematic literature review. *Journal of pain and symptom management* 2011; 41: 1073-1093. 2011/05/31. DOI: 10.1016/j.jpainsymman.2010.08.016.
30. Hareendran A, Skalicky A, Mannix S, et al. Development of a New Tool for Evaluating the Benefit of Preventive Treatments for Migraine on Functional Outcomes – The Migraine Functional Impact Questionnaire (MFIQ). 2018; 58: 1612-1628. DOI: 10.1111/head.13420.
31. Kawata AK, Hareendran A, Shaffer S, et al. Evaluating the Psychometric Properties of the Migraine Functional Impact Questionnaire (MFIQ). *Headache* 2019; 59: 1253-1269. 2019/06/07. DOI: 10.1111/head.13569.
32. Swart ECS, Good CB, Henderson R, et al. Identifying Outcome Measures for Migraine Value-Based Contracting Using the Delphi Method. *Headache* 2020; 60: 2139-2151. 2020/10/01. DOI: 10.1111/head.13978.

For peer review only - http://bmjopen.bmj.com/site/about/guidelines.xhtml

33. Luedtke K, Basener A, Bedei S, et al. Outcome measures for assessing the effectiveness of non-pharmacological interventions in frequent episodic or chronic migraine: a Delphi study. *BMJ Open* 2020; 10: e029855. DOI: 10.1136/bmjopen-2019-029855.
34. Haywood KL AF, Nichols V, Pearce G, Box B, Muldoon L, Patel S, Griffiths F, Stewart K, Underwood M, Matharu M,. Measuring health-related quality of life in chronic headache: a comparative evaluation of the Chronic Headache Quality of Life Questionnaire and Headache Impact Test (HIT-6). *Cephalalgia : an international journal of headache* 2021. DOI: 10.1177/03331024211006045.
35. Staniszewska S, Haywood KL, Brett J, et al. Patient and public involvement in patient-reported outcome measures: evolution not revolution. *Patient* 2012; 5: 79-87. 2012/03/21. DOI: 10.2165/11597150-000000000-00000.
36. Evangelidis N, Tong A, Howell M, et al. International Survey to Establish Prioritized Outcomes for Trials in People With Coronavirus Disease 2019. *Crit Care Med* 2020; 48: 1612-1621. 2020/08/18. DOI: 10.1097/ccm.0000000000004584.
37. Gargon E, Gorst SL and Williamson PR. Choosing important health outcomes for comparative effectiveness research: 5th annual update to a systematic review of core outcome sets for research. *PloS one* 2019; 14: e0225980-e0225980. DOI: 10.1371/journal.pone.0225980.

For peer review only

For peer review only - http://bmjopen.bmj.com/site/about/guidelines.xhtml

Figure 1: Flow diagram outlining the development stages for the COSMIG

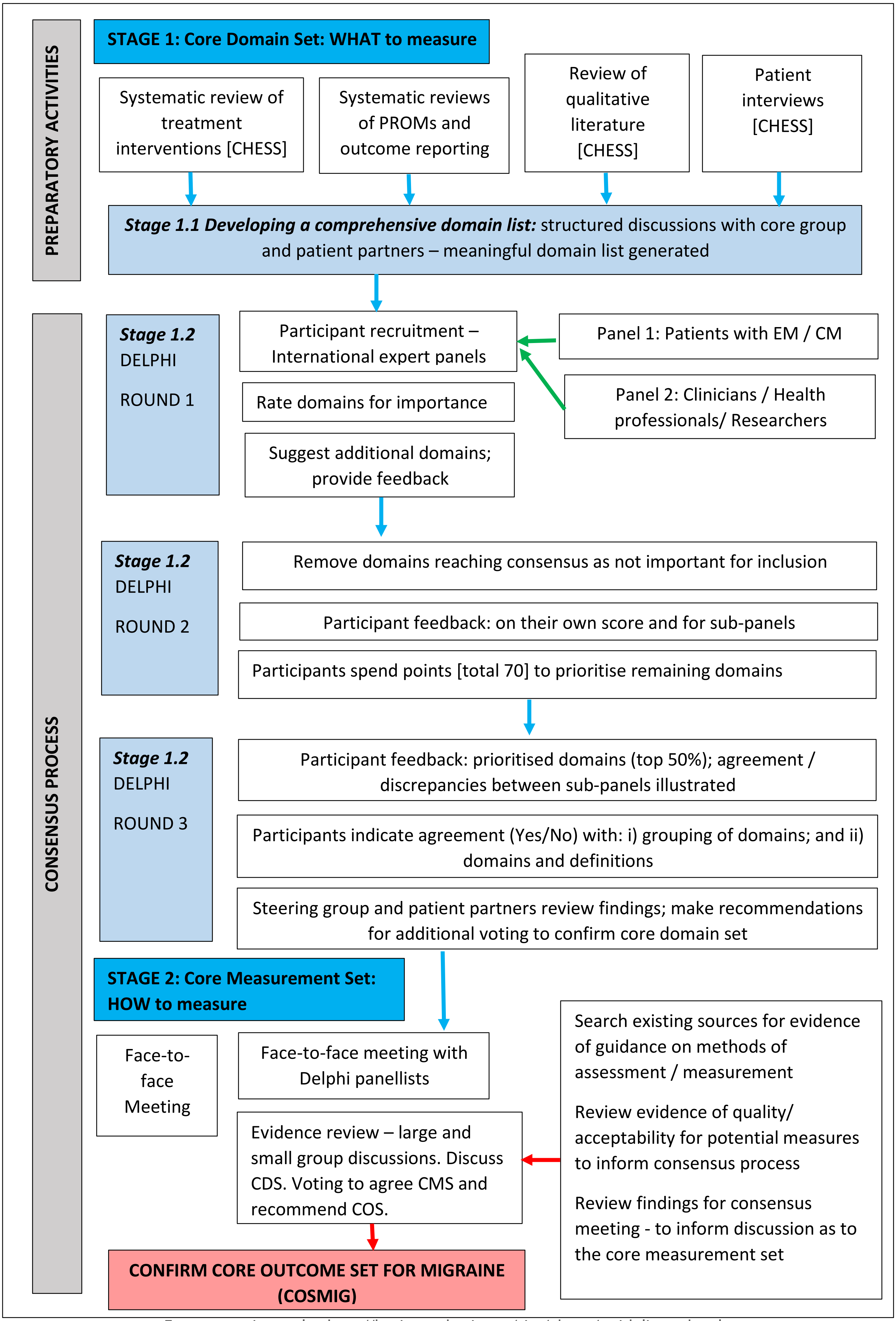

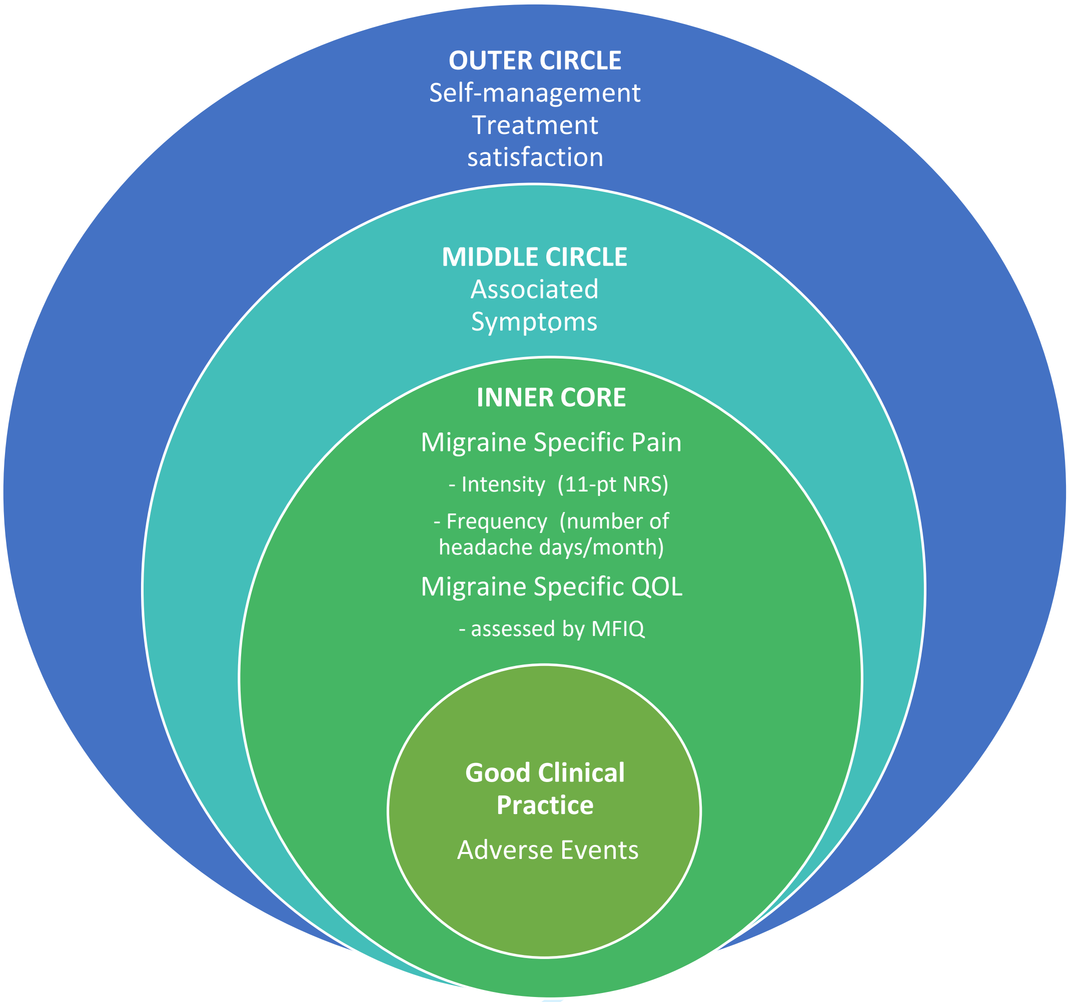

Figure 2. The Core Outcome Set for Episodic and Chronic Migraine (COSMIG):

Footnote: Core 'inner' circle: domain is unambiguous with an acceptable method of assessment;

Middle circle: domain is important, but not feasible for all preventative trials and research studies;

Outer circle: domain is important, but requires further study (research agenda).

For peer review only

APPENDIX

Appendix Table 1. Bespoke grading system to illustrate where consensus was achieved in the Delphi Round 1 for reviewed domains.

Grade	Level of agreement between panel	Decision rule
**A ****	If in both panels the median rating is 9	Include domain in Round 2
**A***	If in both panels ≥70% rate a domain ≥7	Include domain in Round 2
**A**	If in both panels the median domain rating is ≥7	Include domain in Round 2 if either panel achieves a median score of 9 OR qualitative evidence supports further consideration
**B**	If the median rating for a domain is ≥7 in only one panel	Include domain in Round 2 if either panel achieves a median score of 9 OR qualitative evidence supports further consideration
**C**	If the median rating for the two panels combined is ≥4 and ≤6 and the median rating for no single panel is ≤7	No progression to Round 2 (unless qualitative evidence supports further consideration)
**D**	If the median rating for the two panels combined is ≥1 and ≤3 and the median rating for no single panel is ≤7	No progression to Round 2 (unless qualitative evidence supports further consideration)

Footnote: 'both panels' refers to – patient panel and professionals panel

For peer review only - http://bmjopen.bmj.com/site/about/guidelines.xhtml

For peer review only

Appendix Table 2. Background of professional participants (expert panel) in the Delphi process (Round 1).

	Chronic round	Episodic round
Clinician	6	5
Neurologist	13	12
Neurologist specialist interest headache	10	11
GP specialist interest headache	1	0
Nurse specialist	4	3
Chiro/osteopath/	2	1
Health Economist	2	1
Clinical Academic	8	9
Other health professional academic	2	0
Clinical Trialist	9	8
Systematic reviewer	6	5
Measurement expert	7	8

Footnote: participants could identify as having more than one background

For peer review only

For peer review only - http://bmjopen.bmj.com/site/about/guidelines.xhtml

Appendix Table 3. Delphi Round 3: results of voting on sub-panel discrepancies.

Outcome to be voted on (R3)			EPISODIC MIGRAINE *Voting*			CHRONIC MIGRAINE *Voting*		
Discrepancies (outcomes rated in top 50% by one sub-panel)	Proposed Domain and definition	Q	Patient (n=23)	HCPs (n=21)	Combined (n=44)	Patient (n=29)	HCPs (n=23)	Combined (n=52)
Ranked highly by healthcare professionals (HCPs)								
• *HCP 9/27; Patients 20/27 (EM)*	• **Satisfaction with Treatment**	a.	65.2%	**71.4%**	68.2%	-	-	-
• *HCP 8/27; Patients 25/27 (EM)*	• **Vomiting and/ feelings of nausea**	a.	60.9%	**71.4%**	65.9%	-	-	-
• *HCP 12/27; Patients 18/27 (EM)*	• Type (potency) and dose (how much) of a medication when experiencing a migraine	a.				-	-	-
• *HCP 10/31; Patients 20/31 (CM)*	• Stress – feelings of distress, frustration or irritation	a.	-	-	-	58.6%	47.8%	53.9%
• *HCP 15/31; Patients 29/31 (CM)*	• Mortality (death)	a.	-	-	-	20.7%	17.4%	19.2%
Ranked highly by patients								
• *Patients 10/27; HCPs 21/27 (EM)*<br>• *Patients 14/31; HCPs 31/31 (CM)*	• **Unpredictability of a migraine – uncertainty of being symptom-free or able to engage in activities**	a.	**82.6%**	61.9%	**72.7%**	**96.6%**	**69.6%**	**84.6%**
• *Patients 11/27; HCPs 23/27 (EM)*	• **Physical fatigue – experiencing physical fatigue, tiredness, lacking in energy, feeling physically exhausted**	a.	**69.6%**	52.4%	61.4%	-	-	-
• *Patients 10/27; HCPs 21/27 (EM)*	• **Depressive mood – feeling sad, feeling down, feeling sorry for oneself, or feeling depressed**	a.	**69.6%**	42.9%	56.8%	-	-	-

Footnote: Panellists were asked to indicate (Yes/No): a. Should the following outcomes be included in a core set for studies of EM / CM (respectively)?

For peer review only - http://bmjopen.bmj.com/site/about/guidelines.xhtml

For peer review only

COS-START CHECKLIST – FOR COSMIG

SECTION/TOPIC	ITEM NO.	CHECKLIST ITEM	MANUSCRIPT PAGE
**TITLE / ABSTRACT**			
Title	1a	Identify in the title that the paper reports development of a COS	Title: p1
Abstract	1b	Provide a structured summary	Abstract: p2
**INTRODUCTION**			
Background and Objectives	2a	Describe the background and explain the rational for developing the COS	Background: p3
	2b	Describe the specific objectives with reference to developing a COS	Background: p3
Scope	3a	Describe the health condition(s) and population(s) covered by the COS	Background: p3
	3b	Describe the intervention(s) covered by the COS	Background: p3
	3c	Describe the setting(s) in which the COS is to be applied	Background: p3
**METHODS**			
Protocol / Registry entry	4	Indicate where the COS development protocol can be accessed, if available, and /or the study registration details	COMET registration p4
Participants	5	Describe the rationale for stakeholder groups involved in the COS development process, eligibility criteria for participants from each group, and a description of how the individuals involved were identified	Methods: p4 (Stage 1.2)
Information sources	6a	Describe the information sources used to identify an initial list of outcomes	Methods: p4 (Stage 1.1)
	6b	Describe how outcomes were dropped / combined, with reasons (if applicable)	Methods: p4-5 (Stage 1.2);
Consensus process	7	Describe how the consensus process was undertaken	Methods: p4-5 (Stage 1.2); 7 (Stage 1.3)
Outcome scoring	8	Describe how outcomes were scored and how scores were summarised	Methods: p4-5 (Stage 1.2); 7 (Stage 1.3)
Consensus definition	9a	Describe the consensus definition	Methods: p4-5 (Stage 1.2); 7 (Stage 1.3)
	9b	Describe the procedure for determining how outcomes were included or excluded from consideration during the consensus process	Methods: p4-5 (Stage 1.2); 7 (Stage 1.3)
Ethics and consent	10	Provide a statement regarding the ethics and consent issues for the study	Methods: p4
**RESULTS**			

For peer review only

Protocol deviations	11	Describe any changes from the protocol (if applicable), with reasons, and describe the impact these changes have on the results	N/A
Participants	12	Present data on the number and relevant characteristics of the people involved at all stages of COS development	Results: p7-10.
Outcomes	13a	List all outcomes considered at the start of the consensus process	Table 1.
	13b	Describe any new outcomes introduced and any outcomes dropped, with reasons, during the consensus process	Results: p7-10; Tables 1, 2a, 2b, 3, 4, 5
COS	14	List the outcomes in the final COS	Results: p 11; Table 5; Figure 1.
**DISCUSSION**			
Limitations	15	Discuss any limitations in the COS development process	Discussion: p12
Conclusions	16	Provide an interpretation of the final COS in the context of other evidence, and implications for future research	Discussion: p11-12
**OTHER INFORMATION**			
Funding	17	Describe sources of funding / role of funders	Funding statement: p15
Conflicts of interest	18	Describe any conflicts of interest within the study team and how these were managed	Competing interests: p14

For peer review only
